# Enhancing Zinc Bioavailability in Rice Using the Novel Synthetic Siderophore Ligand Proline-2′-Deoxymugineic Acid (PDMA): Critical Insights from Metal Binding Studies and Geochemical Speciation Modeling

**DOI:** 10.1021/acs.jafc.5c02128

**Published:** 2025-03-27

**Authors:** Claudia Rocco, Motofumi Suzuki, Ramon Vilar, Enrique Garcia-España, Salvador Blasco, Gerald Larrouy-Maumus, Colin Turnbull, Matthias Wissuwa, Xuan Cao, Dominik Weiss

**Affiliations:** † Centre for Bacterial Resistance Biology, Department of Life Sciences, Faculty of Natural Sciences, Imperial College London, London SW7 2AZ, United Kingdom; ‡ Department of Earth Science and Engineering, Imperial College London, South Kensington Campus, London SW7 2AZ, United Kingdom; § Aichi Steel Corporation, Tokai-shi, Aichi 476-0003, Japan; ∥ Department of Chemistry, Imperial College London, White City Campus, London W12 0BZ, United Kingdom; ⊥ Instituto de Ciencia Molecular (ICMol), University of Valencia, C/Catedrático José Beltrán Martínez, 2, Paterna 46980, Spain; # Institute of Crop Science and Resource Conservation (INRES), University of Bonn, Karl Robert-Kreiten-Strasse 13, Bonn 53115, Germany

**Keywords:** PDMA, stability constant, fertilization, micronutrient deficiency, mugineic acid, iron

## Abstract

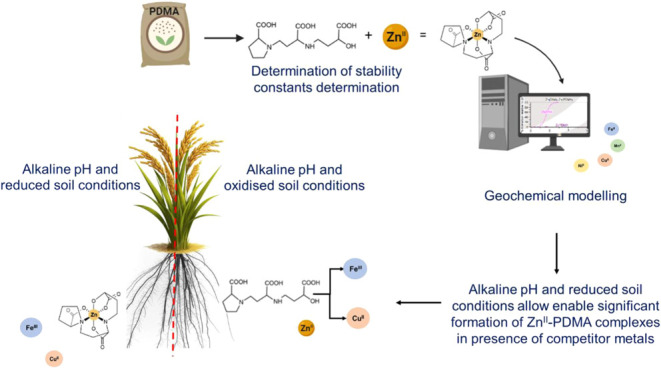

Bioavailable ligands that bind metals mediate their uptake in plants, leading to the study of artificial ligands as potential fertilizers. Proline-2′-deoxymugineic acid (PDMA) has shown a high affinity for Fe^III^, enhancing iron uptake in rice and suggesting that it could be used for improving zinc uptake. This work studied chemical solution parameters, i.e., redox potential, ion strength, pH, and ligand/metal concentrations controlling Zn^II^–PDMA complex formation in rice-producing soils using geochemical speciation modeling. We show that PDMA is generally selective for Zn^II^ in reducing, saline, and alkaline soil solutions. Comparison with a recent micronutrient uptake study in rice suggests that free PDMA should be added in reducing conditions to avoid competition with Cu^II^ and Fe^III^ or as the Zn^II^–PDMA complex at pH below 9. The Zn/M ratios (M = Cu^II^, Fe^III^) needed to form stable Zn^II^–PDMA complexes were also identified. This study shows the promise of PDMA as a fertilizer to overcome zinc deficiencies in alkaline and flooded soils.

## 1 Introduction

Zinc deficiency affects approximately 50% of soils around the globe.^1,2^ In rice growing regions, this deficiency results in low zinc content in rice grains and reduced crop yield^3^ with severe consequences for human health.^4^ Zinc deficiency is becoming rapidly one of the major nutrient disorders in humans, affecting neurobehavioral development, compromising reproductive health, and increasing susceptibility to infectious diseases, including the risk of pneumonia in children.^5−7^

Numerous studies^8−13^ suggest that under zinc-deficient conditions, rice plants exude bioavailable ligands, such as siderophores, able to complex plant-unavailable zinc from the soil matrix and make it plant-available. This is in line with the so-called strategy II mechanism in graminaceous plants^14^ used for the acquisition of insoluble ferric iron (Fe^III^). These ligands are part of the mugineic acid family (MAs; Figure S1). MAs are multidentate amino acids containing three carboxylic acid groups and one azetidine ring able to bind metal ions forming water-soluble octahedral complexes,^15−19^ with the main phytosiderophore secreted by rice being 2′-deoxymugineic acid (DMA).

**Figure 1:**
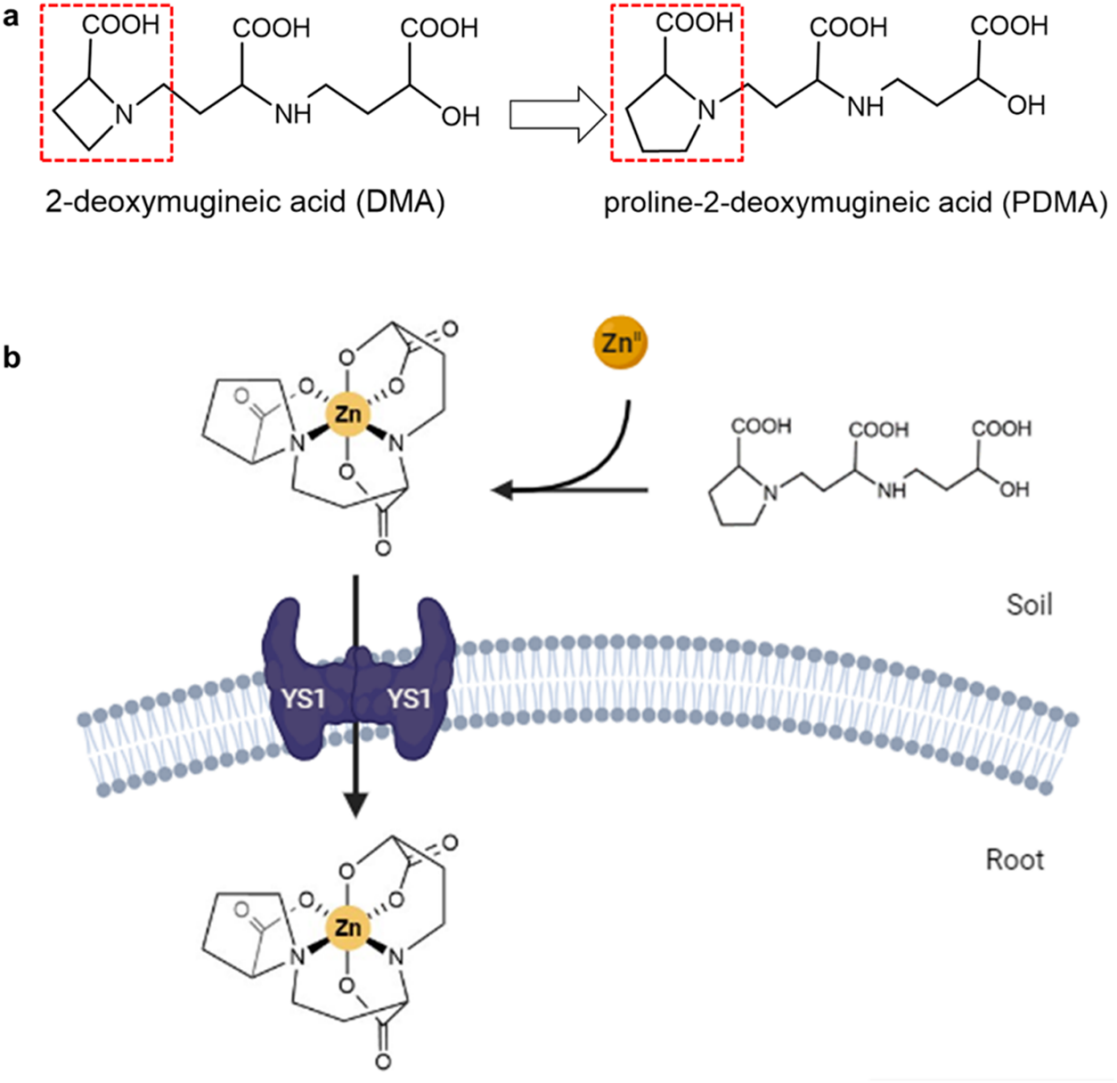
(a) Chemical structure of proline-2′-deoxymugineic acid (PDMA) and 2-deoxymugineic acid (DMA) showing similarity (three carboxylic acid groups and one azetidine ring able to bind metal ions forming water-soluble octahedral complexes) and differences (substitution of the four-membered azetidine ring in DMA to a five-membered ring in PDMA). (b) Schematic representation of mechanisms of how PDMA fertilization would lead to the increased uptake of Zn, i.e., via the suggested formation of a stable Zn^II^–PDMA complex and the subsequent acquisition facilitated by the yellow stripe 1 (YS1) membrane transporter in the root. This mechanism is proposed based on studies of Fe^III^ transport.^19^

Suzuki and co-workers^15^ developed a synthetic analogue to DMA, proline-2′-deoxymugineic acid (PDMA), which has coordinating abilities similar to DMA, but it is easier to synthesize and more resistant to microbial decomposition. It was successfully applied as a fertilizer to increase the uptake of iron in alkaline calcareous soil.^19^ The key difference between the two polydentate ligands is the substitution of the four-membered azetidine ring in DMA to a five-membered ring in PDMA (Figure 1a), improving the resistance to biodegradation and reduced production costs.^15^ Given the structural similarity of DMA and PDMA, it is reasonable to assume that PDMA could be applied to increase the uptake of zinc under deficiency conditions following a proposed mechanism based on Fe^III^ transport studies,^19^ involving complexation of Zn^II^ by PDMA from the soil substrate and the subsequent uptake of the resulting Zn^II^–PDMA complex (Figure 1b). The carboxyl and amino groups of the PDMA ligand are recognized by yellow stripe 1 (YS1) family transporters in an analogous manner to the recognition of naturally occurring DMA.^19^

The critical importance of physicochemical processes in soil solutions during micronutrient acquisition is well recognized.^20,21^ Soil solution refers to soil water, including dissolved solutes, and was described as the “blood circulation of the soil body”.^20^ Chemical reactions, including metal–ligand (M–L) complexation, occur in or are mediated by the soil solution. Chemical parameters, including pH, redox potential, and ion strength, control formation and stability of M–L complexes and hence the potential of PDMA to be used as a fertilizer.^22−25^ Furthermore, competitive complexation with other bi- and trivalent metal cations present in solutions is critical, with concentrations depending greatly on the composition of the soil parent material and on anthropogenic activities.^26−28^

If PDMA is to be used as a zinc fertilizer, it is therefore critical to identify the effect of chemical solution parameters on the stability of Zn^II^–PDMA complexes in circumstances relevant to rice production. Rice is cultivated in a wide range of soil conditions, with the pH ranging from acidic to alkaline (typically 4 to 9, depending on bedrock), redox potential ranging from oxidizing/aerobic to reducing/anaerobic (typically 700 to −300 mV, related to aerated and waterlogged conditions), and ionic strength typically ranging from dilute to slightly saline (0.02 to 0.16 mol/dm^3^).^16,17^

Experimental investigations of soil solution reactions, especially in the microenvironment around plant roots, are extremely difficult to conduct. To this end, speciation calculations are increasingly applied to perform a wide variety of aqueous geochemical modeling.^11^ Numerous linear and nonlinear thermodynamic mass balance expressions are solved simultaneously, and geochemical speciation models are developed to study how chemical species behave in complex soil solutions under changing environmental conditions.^29^

Here, we present results from a combined experimental and modeling study aiming to constrain metal binding and ion interaction of proline-2′-deoxymugineic acid (PDMA) with trace metals in solutions pertinent to soils employed for rice production with the aim to assess the possible role of this synthetic siderophore to overcome zinc deficiency in rice. To this end, we performed the following investigations.

(i) We determined proton and metal–ligand (M–L) stability constants for PDMA with Zn^II^, Fe^II^, Fe^III^, Cu^II^, Co^II^, Ni^II^, Mg^II^, and Mn^II^ and developed accurate chemical models for the M–PDMA systems studied.
(ii) We investigated the effect of major chemical solution parameters (pH, ion strength, redox potential, competition) on the formation of M–PDMA complexes in aqueous solutions.
(iii) We investigated the link between PDMA speciation in soil solutions and the uptake of zinc and other trace metals in rice using a recent study,^15^ testing the effect of PDMA fertilization on nutrient uptake in rice.
(iv) We identified the range of excess iron and copper in soil solutions tolerable to not interfere with the formation and stability of Zn^II^–PDMA complexes and hence ensure the efficiency of the fertilizer.

The geochemical model developed and used in this study includes solution processes only, and the effects of other important soil processes including interactions with minerals and organic phases at the solid–solution interface or microbial activities need to be tested to confirm the robustness of our recommendations.

## 2 Materials and Methods

### 2.1 Chemicals

Tris(hydroxymethyl)aminomethane (THAM, Roche Diagnostics) was used to calibrate 0.1 mol/dm^3^ HCl solutions used for the potentiometric titrations. CO_2_-free NaOH standard solutions (0.1 mol/dm^3^) were supplied by VWR and protected from atmospheric CO_2_ by means of soda lime traps. NaCl (0.15 mol/dm^3^) was used as an electrolyte solution during potentiometric analysis. Proline-2′-deoxymugineic acid (PDMA) was provided by Aichi Steel Company (Japan). Unless otherwise stated, the purity of all chemicals was ≥97% (on a mass basis), and they were used without further purification. Details on the synthesis and characterization of PDMA are described by Suzuki et al.^15^ Metal ion (Zn^II^, Fe^II^, Fe^III^, Cu^II^, Co^II^, Ni^II^, Mg^II^, and Mn^II^) solutions (0.1 mol/dm^3^) were prepared by dissolving the corresponding mass of metal salts in ultrapure water. Specifically, ZnCl_2_ (anhydrous, VWR), MgCl_2_·6H_2_O (Sigma-Aldrich), MnCl_2_ (Sigma-Aldrich), FeSO_4_·6H_2_O (Sigma-Aldrich), FeCl_3_·6H_2_O (Sigma-Aldrich), CuCl_2_ (extra pure, Thermo Scientific), NiCl_2_·6H_2_O (Sigma-Aldrich), and CoCl_2_·6H_2_O (BDH) were used for the determination of logK values for the PDMA–metal and PDMA–H complexes. Titrations with (ethylenediaminetetraacetic acid (EDTA), Fisher Scientific) were done to determine the effective molar concentration of all metal solutions. Ultrapure water (18 MΩ cm^–1^), grade A glassware, and analytical grade reagents were used throughout.

### 2.2 Experimental Determination of Ligand and Proton Stability Constants

Potentiometric measurements were conducted in an inert atmosphere by bubbling purified nitrogen through solutions at *T* = 298.1 ± 0.1 K using a thermostatic cell. A Metrohm automatic titrator Titrando 888 controlled by Metrohm TiAMO version 2.3 software was used for the potentiometric titrations of PDMA to determine ligand and proton stability constants. At least two measurements were performed to minimize systematic errors and to check the repeatability of the measurements. The hydrogen ion concentration was measured with a combined pH glass electrode.

A 30 mL solution containing PDMA and NaCl was titrated with standardized NaOH solutions. Ligand concentrations were slightly in excess, with respect to the metal concentration, to avoid metal hydrolysis interference during the analysis. In general, the titrant solutions consisted of 6.7 × 10^–4^ mol/dm^3^ of PDMA and 6.0 × 10^–4^ mol/dm^3^ of the metal studied. All of the measurements were performed under magnetic stirring in thermostat cells using an isothermal bath.

For each experiment, independent titrations of strong acid solutions with standard strong base solutions were carried out under the same medium and ionic strength as the systems to be investigated, with the aim of determining the reference electrode potential (*E*^0^) using GLEE software^30^ (Version 3.0.21). For each titration, 80 to 100 data points were collected, and the equilibrium state during titrations was checked by confirming the time required to reach equilibrium. All the potentiometric titrations were made over the pH range of 3–10.

**Figure 2:**
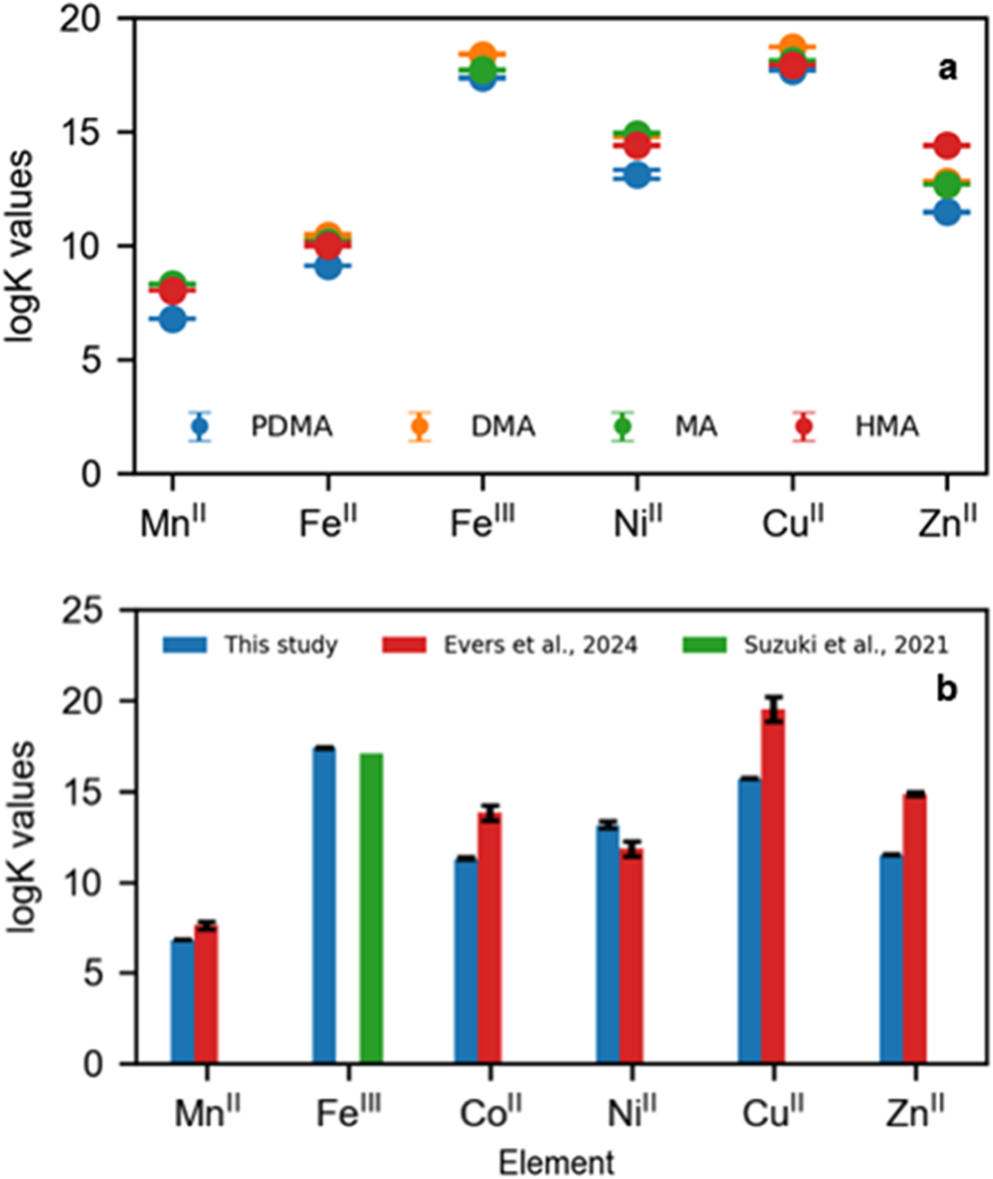
(a) Logarithm of stability constants (log *K*) of selected transition row metals with PDMA (this study), MAs (mugineic acid family, i.e., DMA, MA, and HMA, Murakami et al.^31^; Weiss et al.^13^). (b) The logarithm of stability constants of metal–PDMA complexes determined by Evers et al.,^44^ Suzuki et al.,^15^ and during this study. PDMA = proline-2′-deoxymugineic acid, DMA = 2′ deoxymugineic acid, MA = mugineic acid, HMA = 3-epi-hydroxy-mugineic acid. PDMA log *K* values in this study were determined at *I* = 0.15 mol/dm^3^ in NaCl and *T* = 298.1 K using a 1:1 M/L molar ratio. Evers et al. used spectrophotometric titration at *I* = 0.1 mol/dm^3^ NaCl or NaNO_3_ and a 1:1 M/L molar ratio. DMA, MA, and HMA were determined at *I* = 0.1 M in KNO_3_ and *T* = 298.1 K and 1:1 M/L molar ratio.

Hyperquad Simulation and Speciation software (HySS, version 4.0.31) was used to determine experimental conditions for the titrations, identify the chemical model, and calculate the stability constants from the potentiometric data set. Examples of simulated titrations for the PDMA and Zn^II^–PDMA systems in HySS are shown in Figure S2. The titration curves of repeated titrations for each system were treated in Hyperquad as a single set when refining the stability constants (Figure S3). This means that the refinement procedure was run on both replicated curves at the same time to derive a single set of constants. The error reported for the stability constants is the 1 sigma standard deviation. Figure 2 and Table S1 list the conditional log *K* values for PDMA/metal complexes and MAs/metal complexes as taken from the literature.^31^

### 2.3 Thermodynamic Speciation Calculations and Geochemical Modeling

Thermodynamic speciation calculations for M–PDMA systems were conducted using HySS (see above) and PHREEQC, a computer program designed to perform aqueous geochemical calculations (v. 3.7.3.15968).

HySS was used for calculating the speciation of M–L systems (3.1) and to examine competitive binding (paragraph 3.2). We used the conditional stability constants determined during this study. Concentrations of species were calculated based on a given chemical model and pH range.^32^ The speciation calculations were conducted using [*M*] = 10^–6^ mol/dm^3^ and [*L*] = 10^–5^ mol/dm^3^, which is the standard procedure adopted for studying *M*–*L* interactions in aqueous solutions.^23,33,34^ The sensitivity of our speciation calculations is estimated to be 5% for the concentration of 1 mol/dm^3^ and 0.5% for the concentration of 0.05 mol/dm^3^.^23^

PHREEQC was used for conducting computational experiments (i) to examine the role of chemical solution parameters including pH, ion strength, and redox potential on *M*–*L* stability (paragraph 3.2), (ii) to determine M–PDMA complex formation in the soil solutions of the plant uptake experiments conducted by Suzuki et al.^15^ (paragraph 3.3), and (iii) to identify the limit of excess Cu and Fe admissible in soil solution to maintain stable Zn^II^–PDMA complexes and hence to identify soil solution conditions where PDMA can be used as a zinc fertilizer (paragraph 3.4).

Our model development has been described in detail elsewhere.^11,23,29^ PHREEQC has 13 databases containing thermodynamic properties of organic and inorganic *M*–*L* complexes, and following evaluation for consistency and completeness, we used the minteq database as default and added the new M–PDMA stability constants determined during this study. Stability constant values (log *K*) determined experimentally in a 0.1 mol/dm^3^ NaCl electrolyte solution were converted into intrinsic stability constants using the Davies equation (eq 1)^23,35^
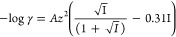
1where *A* is the dielectric constant of the solvent (*A* = 0.51 at *T* = 298.1 K), *z* is the charge of the ion, and *I* is the ion strength (mol/dm^3^). PHREEQC uses pe to define redox potential, and it is related to Eh values by eq 2.^36^
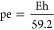
2

The selectivity of PDMA for Zn^II^ compared to the other metals (M) assessed was calculated using eq 3
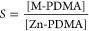
3where [M-PDMA] and [Zn^II^-PDMA] are the concentrations of *M*–*L* complexes. Since S can span orders of magnitude, we report it as a logarithmic value (i.e., log *S*). If log *S* > 0, PDMA is more selective for M, and if log *S* < 0, PDMA is more selective for Zn^II^.

Model accuracy was evaluated by running selected experiments using HySS and PHREEQC models and comparing the model’s predictions (slope: 0.9968; *R*^2^ = 0.9936; Figure S4).

### 2.4 Computational Experiments

Computational experiments were conducted (i) to identify the constraints of chemical solution parameters (pH, redox potential, ionic strength) and competition (ligands, metals) on M–PDMA complex formation (Section 3.2), (ii) to determine metal speciation in soil solutions of previous fertilization experiments to enhance micronutrient uptake in rice using PDMA^21^ (Section 3.3), and (iii) to identify Cu and Fe excess permissible in soil solution to sustain stable Zn^II^–PDMA complexes (paragraph 3.4).

pH was typically varied from 3 to 9 to account for acidic and alkaline soil solutions, ionic strength (*I*) was varied between 0.02 and 0.7 mol/dm^3^ to account for the extent of salinity, and Eh was varied between −300 and +350 mV to account for reduced and oxidized soil conditions. Rice is typically cultivated in lowland flooded soils.^37−39^ These soils are drained during the tillering stage and immediately before the harvest to enable faster rice ripening and harvesting.^37,40^ Flooding and draining periods affect the redox chemistry, leading to substantial changes in the chemical composition of the soil solution and thus critical regarding the effectiveness of PDMA as a fertilizer.^37,40^

**Figure 3:**
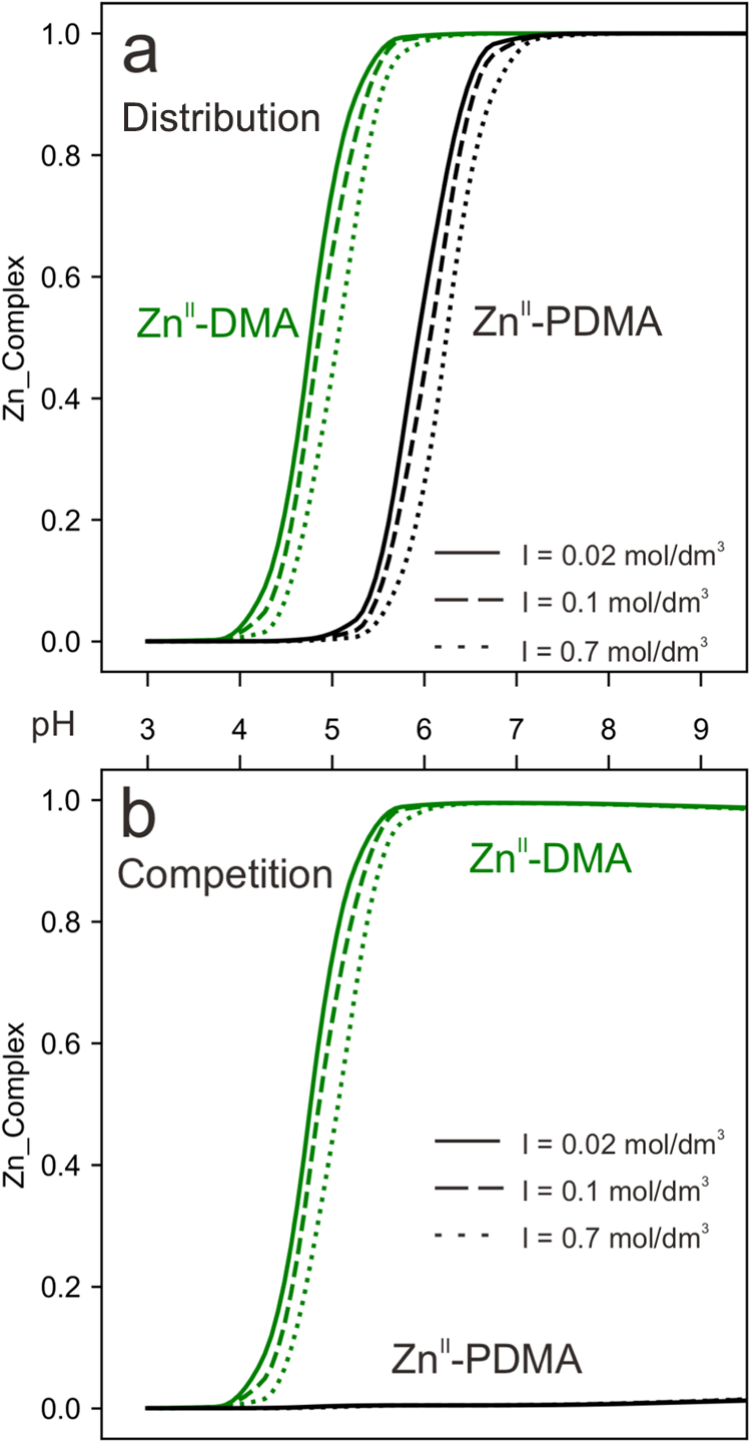
Fraction of complexed Zn^II^ with PDMA and DMA as a function of the pH at different ionic strengths in single (a) and mixed (b) solutions. Ion strengths (*I*) = 0.02, 0.1, and 0.7 mol/dm^3^. The concentrations used were 10^–6^ mol/dm^3^ for Zn^II^ and 10^–5^ mol/dm^3^ for PDMA and DMA. Different types of lines represent different ionic strengths.

In Section 3.2, speciation calculations used a molar ratio of 1:10 for *M*/*L* with [*M*] = 1 × 10^–6^ mol/dm^3^. For the competitive binding experiments, the trace metals studied were Zn^II^, Fe^II^, Fe^III^, Cu^II^, Co^II^, Ni^II^, Mg^II^, and Mn^II^ (Figure 5). The organic ligands studied were PDMA, DFOB (desferrioxamine B), and DMA representing strongly binding ligands, and citrate, oxalate, and malate representing weak binding ligands (Figures 3, 6, and S5). The aim was to assess how they compete for complexation, as these organic ligands are commonly present in soil exuded by plants or microorganisms such as bacteria, ectomycorrhizal, and lichenous fungi.^22,23,41^

In Section 3.3, we used the chemical composition of the nutrient solution applied by Suzuki et al.^15^ during a fertilization study testing the effect of PDMA addition on micronutrient uptake in rice. The solution composition was 0.5 mmol/dm^3^ MgSO_4_, 0.5 μmol/dm^3^ MnSO_4_, 0.2 μmol/dm^3^ CuSO_4_, 0.5 μmol/dm^3^ ZnSO_4_, and 0.1 mmol/dm^3^ Fe-EDTA. The results of our speciation models were then compared with metal content in rice from control and fertilization experiments. During the pot experiment, rice (*Oryza sativa*, var. Nipponbare) plants were transplanted into calcareous soil (pH 9). 14 days after transplanting, rice soil (Eh = 350 mV) was treated with free PDMA and with PDMA complexed with Fe^III^ and Zn^II^. The soil was watered 3 times a week until saturated water capacity.^15^ Harvest of plants was after 70 days, and whole plants were analyzed for Cu, Zn, Fe, and Mn.

In Section 3.4, Zn/(Fe, Cu) and (Fe, Cu)/Zn molar ratios ranged from 2 to 10 and from 2 to 50, respectively, with initial Zn/(Fe, Cu) ratios based on the average concentration of zinc, copper, and iron present in rice soils.^28,42^ The NaCl electrolyte solution used was 0.1 mol/dm^3^. The range of pH and Eh aimed to cover acidic, neutral, and alkaline and reduced and oxidized soil solutions found in different rice-producing soils. Fe^II^ was not included because no complex formation with PDMA occurs due to its lower log *K* value compared with Zn^II^ and Cu^II^.

## 3 Results and Discussion

### 3.1 Proton and Metal Association Constants for PDMA and pH-Dependent Speciation

We first conducted potentiometric titrations to determine proton and metal–ligand (M–L) stability constants for PDMA with Zn^II^, Fe^II^, Fe^III^, Cu^II^, Co^II^, Ni^II^, Mg^II^, and Mn^II^ in 0.15 mol/dm^3^ NaCl electrolyte solutions and to establish accurate chemical models for the different M–PDMA systems studied.

#### 3.1.1 Protonation (log *K*_a_) and Metal Association (log *K*) Constants for PDMA

Table 1 shows protonation constants (log *K*_a_) for PDMA. The fully protonated PDMA has a charge of +2 (H_5_PDMA^2+^), while fully deprotonated PDMA has a charge of −3 (PDMA^3–^). Both PDMA^3–^ and HPDMA^2–^ chelate divalent metals, yielding complexes with net charges of −1 and 0, respectively. Three of the five protonation constants of PDMA were determined, as the last two protonation steps (H_4_PDMA^+^ and H_5_PDMA^2+^) happen at a pH too low to be accurately determined by potentiometric titrations. The protonation constant values of H_3_PDMA, H_2_PDMA^–^, and HPDMA^2–^ (Table 1) are in agreement with Suzuki et al.^15^ who determined log *K*_a_ values by potentiometric titrations in 0.1 mol/dm^3^ KNO_3_ and *T* = 293.15 K. These three protonation steps occur on the two amines and one of the carboxylate groups. Titration curves and manually fitted models, chemical speciation models, and the experimental conditions are given in the Supporting Information (Figures S2 and S3).

**Table 1:** Protonation Constants (log *K*_a_) of PDMA Determined in 0.15 mol/dm^3^ NaCl Solution and *T* = 298.1 K (This Study) and in 0.1 mol/dm^3^ KNO_3_ and *T* = 293.1 K by Suzuki et al.^a^

	log *K*_a_	
reaction	this study	Suzuki et al.
	10.32 ± 0.01	9.23
	8.08 ± 0.01	7.63
	2.66 ± 0.02	3.12
		2.47
		2.01

a For this study, determinations were conducted in triplicate and the error was ± σ. No errors were reported for Suzuki et al.

Table 2 shows log *K* values for the complexation of PDMA with the metal ions evaluated and the species formed. Next to Zn^II^, Fe^II^, Fe^III^, Cu^II^, Ni^II^, Mn^II^, Mg^II^, and Co^II^ were chosen because of their potential to compete for PDMA in soil solutions and their importance in agricultural soils and in plant growth and development.^27^ They are also present in the formulation of mineral fertilizers used in agriculture.^26^ Dominant species are shown in different columns in Table 2 and refer to distribution diagrams in Figure S2.

**Table 2:** Stability Constants (log *K*) for Complexation of Zn^II^, Fe^II^, Fe^III^, Cu^II^, Co^II^, Ni^II^, Mg^II^, and Mn^II^ with PDMA Determined Potentiometrically in 0.15 mol/dm^3^ NaCl and *T* = 298.1 K^a^

	log *K*		
metal	M + L ⇆ ML	ML + H ⇆ MHL	ML + H_2_O ⇆ ML(OH) + H
Zn^II^	11.48 ± 0.02		–9.69 ± 0.03
Fe^II^	9.11 ± 0.01	6.20 ± 0.02	–9.73 ± 0.01
Fe^III^	17.37 ± 0.02	2.53 ± 0.01	
Cu^II^	17.29 ± 0.03		–9.93 ± 0.05
Co^II^	11.27 ± 0.07		–9.74 ± 0.29
Ni^II^	13.10 ± 0.2	3.62 ± 0.14	–9.96 ± 0. 03
Mg^II^	3.69 ± 0.05	9.26 ± 0.12	–10.35 ± 0.03
Mn^II^	6.81 ± 0.005	6.9 ± 0.04	–9.68 ± 0.01

a Determinations were conducted in triplicate and the error is ± σ.

The log *K*_ML_ value for the formation of [Zn(PDMA)]^−^ is 11.48 ± 0.03 (Zn^2+^ + PDMA^3–^ ⇌ [Zn(PDMA)]^−^). The complex formation starts at around pH 4 and is quantitative at pH 6. [Zn(PDMA)(OH)]^2–^ forms significantly at pH above 9, with a log *K*_MLOH_ value of −9.69 ± 0.03 (Zn^2+^ + H_2_O + PDMA^3–^ ⇌ [Zn(PDMA)(OH)]^2–^ + H^+^). PDMA therefore forms stable complexes with Zn^II^ in the pH range found in soils, i.e., between 4 and 9.

PDMA forms important 1:1 M/L complexes with the other metals studied, with Mg^II^, forming the weakest complex (log *K*_ML_ = 3.69 ± 0.05), and Fe^III^, forming the strongest complex (log *K*_ML_ = 17.37 ± 0.02; Table 2 and Figure 2a). Below pH 4, we find M/L/H species for Fe^II^, Fe^III^, Ni^II^, Mg^II^, and Mn^II^; while at pH above 9, we identify M:L:OH species for Cu^II^, Co^II^, Ni^II^, Mn^II^, and Mg^II^. The trend for the formation of M^II^–PDMA complexes follows the Irving–Williams series.^43^
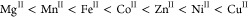
Cu^II^ and Fe^III^ will compete with Zn^II^ for PDMA^3–^, while Mg^II^ and Mn^II^ are unlikely to be significant competitors.

Figure 2b summarizes stability constants for M–PDMA complexes determined during this and previous studies.^15,44^ The log *K*_ML_ values reported by Evers et al.^44^ (Table S1) are higher than those determined in this study, with the exception of Ni^II^. The largest differences are for log *K*_ML_ values with Zn^II^ (11.48 ± 0.02 vs 14.85 ± 0.1) and Cu^II^ (15.69 ± 0.03 vs 19.5 ± 0.7). These variations could reflect the different analytical techniques and procedures employed. Evers et al.^44^ used spectrophotometric titration in 0.1 mol/dm^3^ NaCl and NaNO_3_ solutions with a 1:1 molar ratio for M/L. The stability constant for the Fe^III^–PDMA complex determined by Suzuki et al.^15^ (Table S1) using potentiometric titration in 0.1 mol/dm^3^ KNO_3_ with a 1:1 molar ratio for M/L is in line with our results (17.1 vs 17.32 ± 0.02).

#### 3.1.2 pH-Dependent Formation of M–PDMA and M–MAs Complexes

Table S1 lists stability constants for selected metals with bioavailable ligands from the mugineic acid family, including mugineic acid (MA), 2′-deoxymugineic acid (DMA), and 3-epi-hydroxy-mugineic acid (HMA). Figure 2a displays log *K*_ML_ values for the M–PDMA and M–MAs complexes. The trend between metals is similar for all M–L complexes studied, i.e., MA, DMA, and HMA.^45^ The log *K*_ML_ values for Mn^II^, Ni^II^, Fe^III^, Cu^II^, and Zn^II^ are consistently lower for PDMA compared to MAs, i.e., between 0.36 to 0.83 log *K* units.

### 3.2 Effect of Chemical Solution Parameters on Zn^II^–PDMA Formation

In a second step, we studied the effect of master chemical solution parameters distinctive for rice-producing soils on the formation of metal–PDMA complexes, i.e., ion strength, redox potential, and competition.

#### 3.2.1 Ionic Strength

Figure 3a shows the pH-dependent fraction of Zn^II^ complexed to PDMA and DMA at low, medium, and high ionic strengths. i.e., 0.02, 0.1, and 0.7 mol/dm^3^, respectively. At low ion strength, the formation of Zn^II^ –PDMA starts at pH 5 and is quantitative at pH 7. At medium and high ion strengths, the formation of Zn^II^–PDMA complexes shifts slightly toward higher pH values, reaching quantitative complexation at pH 7 and 7.5, respectively. DMA exhibits a similar pH-dependent pattern for complex formation with Zn^II^. This is expected as the chemical structure of DMA and PDMA are similar. The formation of Zn^II^–DMA complexes starts at around pH 4 instead of pH 5 for Zn^II^–PDMA. As observed for PDMA, the formation of Zn^II^–DMA complexes shifts to the higher pH range as a consequence of the increase in ionic strength.

Figure 3b shows the competition between DMA and PDMA for Zn^II^ complexation when both ligands are present at equal concentrations, i.e., molar ratio of Zn^II^/PDMA/DMA = 1:10:10. Under these conditions, Zn^II^ is complexed by DMA, as log *K*_ML_ values are higher (Figure 2 and Tables 1 and S1).

#### 3.2.2 Redox Potential

**Figure 4:**
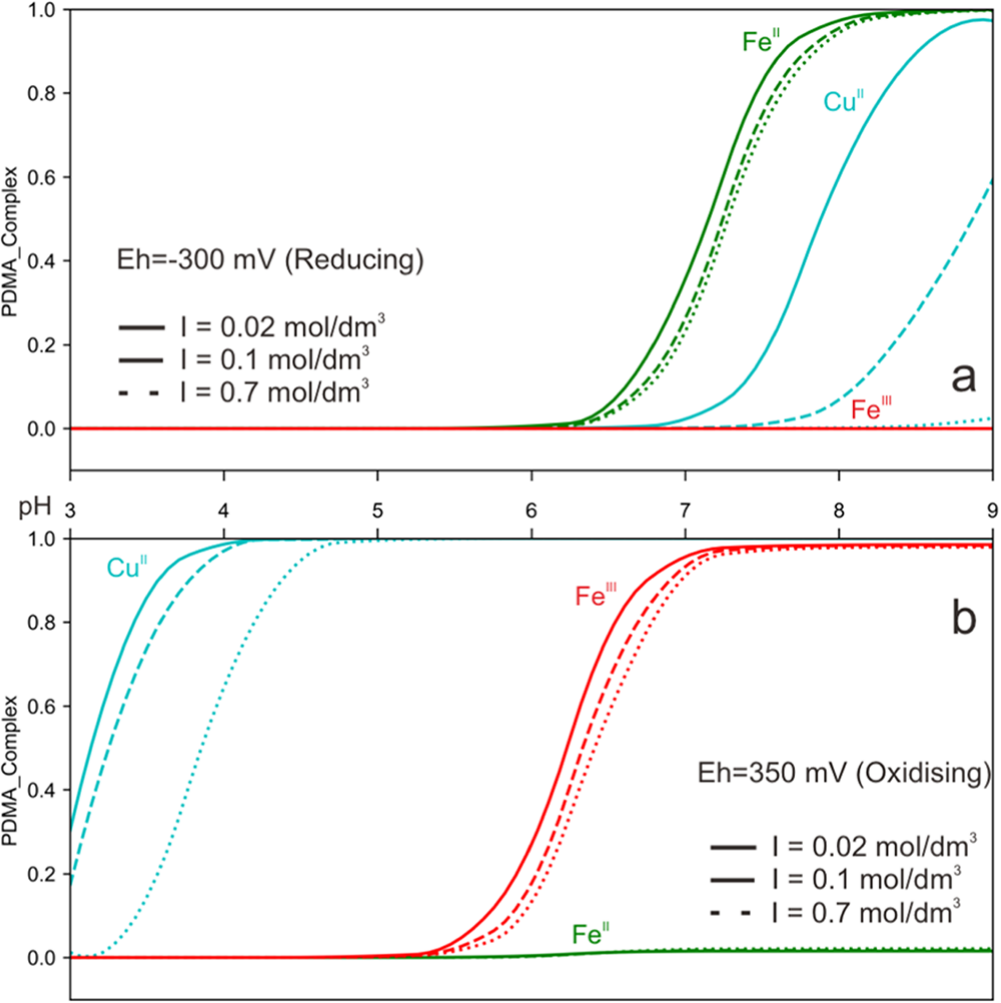
Fraction of complexed metal (Cu^II^, Fe^II^, and Fe^III^) as a function of pH in NaCl solutions with different ionic strengths (*I* = 0.02, 0.1, and 0.7 mol/dm^3^) and redox conditions (a): Eh = −300 mV; (b): Eh = +350 mV. The concentrations used in the model are 10^–6^ mol/dm^3^ for metals and 10^–5^ M for PDMA. Different types of lines represent different ionic strengths. Mn^II^ is redox-sensitive but does not form strong complexes with PDMA due to its low log *K* value, and for that reason, it is not included in the figure.

The oxidation state of iron and copper is controlled by the redox potential of the solution, and changes will affect the complexation capacity of Zn^II^ with PDMA.^25^Figure 4 shows the fractions of Cu and Fe complexed to PDMA as a function of the pH in solutions with different ion strengths and redox potential.

In solutions with negative redox potential, copper is present as Cu^I^ and significant complexation takes place only at pH above 6.8 and at low ionic strength. At pH above 7, copper is partially present as copper(II)hydroxide, [Cu(OH)]^+^.^25^ The formation constant for Cu^II^ with PDMA is far larger than with OH^–^ (log *K*_ML_ 17.29 and −7.42), explaining why at negative redox potentials, [Cu(PDMA)]^−^ complexes are formed.

In solutions with positive redox potential (Figure 4b), approximately 30, 17, and 1% of Cu^II^ is complexed with PDMA at pH 3 and at ion strengths of 0.02, 0.1, and 0.7 mol/dm^3^, respectively, reaching quantitative complexation at around pH 4.5 for low- and medium-ionic-strength solutions and at pH 5 for high-ionic-strength solutions. Comparable results are obtained for iron. In reducing solutions (Eh = −300 mV, Figure 4a), iron is present in its reduced form,^25^ and PDMA complexation with Fe^II^ starts approximately at pH 6 and reaches 83, 75, and 71% complexation at ionic strengths of 0.02, 0.1, and 0.7 mol/dm^3^, respectively. At pH 8.5, iron is complexed quantitatively by PDMA. In oxidizing solutions (Eh = +350 mV, Figure 4b), iron is oxidized and PDMA complexation starts approximatively at pH 5.5; 96, 93, and 91% of iron is complexed in solutions of low, medium, and high ionic strengths, respectively. Only 2% of total iron remains dissolved in the aqueous solution as iron oxides are formed.^25^

#### 3.2.3 Competitive Binding of Metals

**Figure 5:**
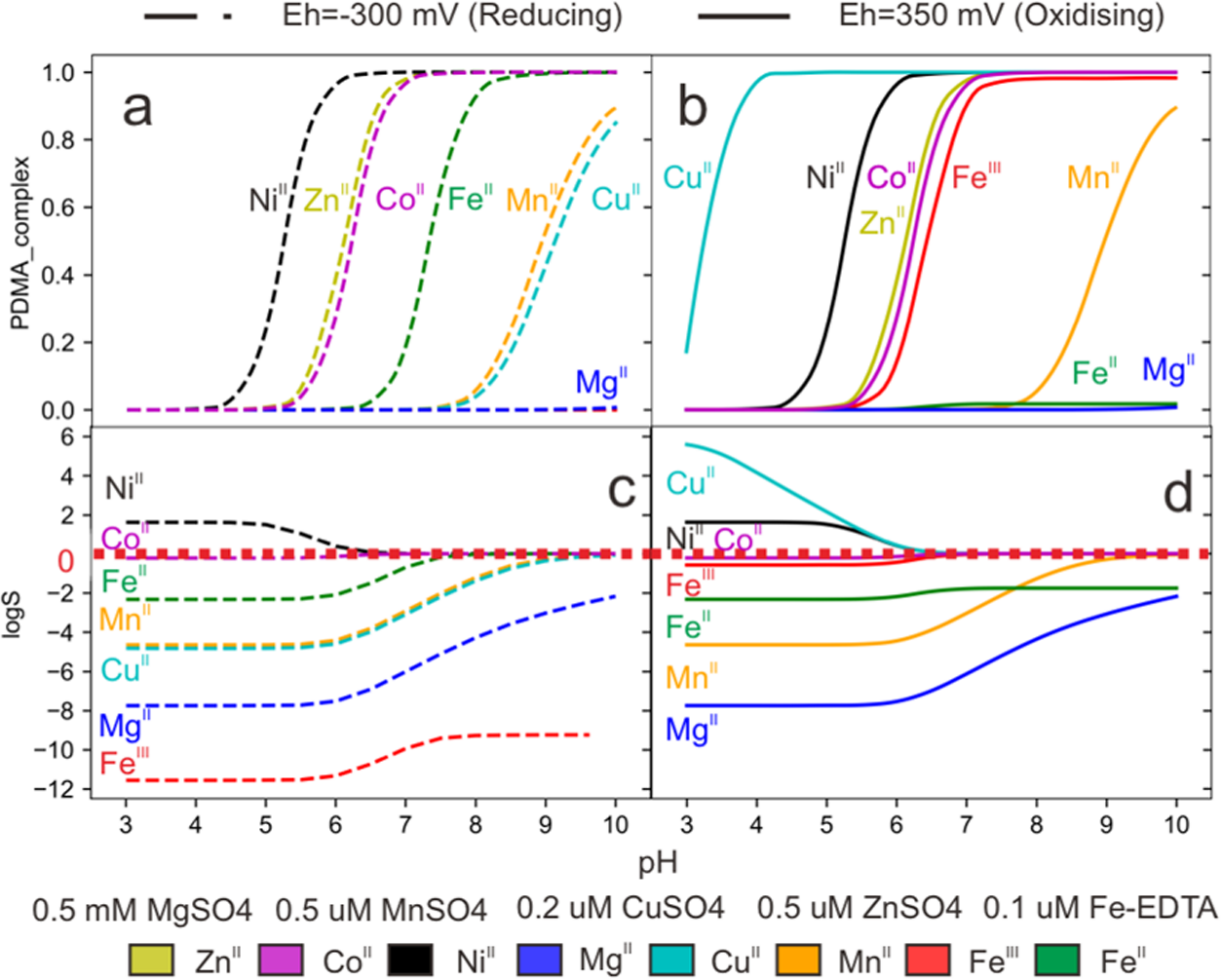
Fraction (a, b) of metal–PDMA complexes with Zn^II^, Fe^II^, Fe^III^, Ni^II^, Cu^II^, Mg^II^, Mn^II^, and Co^II^, and selectivity (c, d) of metal–PDMA relative to Zn^II^–PDMA, expressed using the log of the selectivity ratio (log *S*, metal/Zn^II^), as a function of pH in different redox conditions (a, c): Eh = −300 mV; b, d: Eh = +350 mV at 0.1 mol/dm^3^ ionic strength. The concentrations used were 10^–6^ mol/dm^3^ for metals and 10^–5^ mol/dm^3^ for PDMA.

Figure 5 displays pH-dependent (i) formation of PDMA complexes with the metals studied (Zn^II^, Fe^II^, Fe^III^, Ni^II^, Cu^II^, Mg^II^, Mn^II^, Co^II^) and (ii) selectivity for the formation of Zn^II^–PDMA over that of other M^II,III^–PDMA complexes in reducing and oxidizing solutions, i.e., with positive and negative redox potentials (Figure 5a–c: Eh = −300 mV; Figure 5b–d: Eh = +350 mV).

Under reducing conditions, iron is present as ferrous iron, and no PDMA complex with Fe^III^ forms (Figure 5a). Mg^II^ does not form complexes with PDMA at any pH due to its low logK_ML_ value. In acidic solutions, PDMA forms complexes with Ni^II^, Zn^II^, and Co^II^ (pH between 4.5 to 5.5). In near-neutral solutions (pH 6.5), PDMA forms complexes with Fe^II^. In the pH range between 7.5 and 8, PDMA complexes with Mn^II^ and Cu^II^, indicating that in alkaline solutions, significant M^II^–PDMA complexes are formed even with metals with low log *K*_ML_. Mn^II^ and Mg^II^ do not compete with Zn^II^ for PDMA at any point, i.e., log *S* < 0, due to the significantly lower log *K*_ML_ of these complexes (Figure 5c). log *S* is zero for Ni^II^ and Co^II^ above pH 6.5, for Fe^II^ above pH 8, and for Fe^III^ above pH 9. Below pH 6.5, Ni^II^ is the only competitor for Zn^II^ (log *S* > 0). Copper is present in the reduced form (Cu^I^), and therefore, no Cu–PDMA complexes are formed.

Under oxidizing conditions, the speciation of Ni^II^, Mg^II^, Mn^II^, Co^II^, and Zn^II^ is the same as under reducing conditions (Figure 5b). Ni^II^ and Zn^II^ are stable in their divalent forms under the investigated redox conditions. A similar behavior is detected for Mn^II^ and Co^II^, as confirmed by the literature.^2,25^ In contrast, the speciation of Cu^II^, Fe^III^, and Fe^II^ is different, as seen in Figure 4. Cu^II^ complexation starts at a very low pH (pH < 3), and Fe^III^ complexation starts at around pH 5.5. Only 2 and 1% of Fe^II^ and Mg^II^ are complexed in alkaline solutions (Figures 4b, and 5b). logS values are negative for Mg^II^ and Fe^II^ across the pH range studied. (Figure 5d). At pH values below 6.5, the selectivity of PDMA increases for Cu^II^ and Ni^II^, with logS values of 5.6 and 1.6, respectively. At this redox potential, iron precipitates.^25^

#### 3.2.4 Competitive Binding of Strong and Weak Organic Ligands

**Figure 6:**
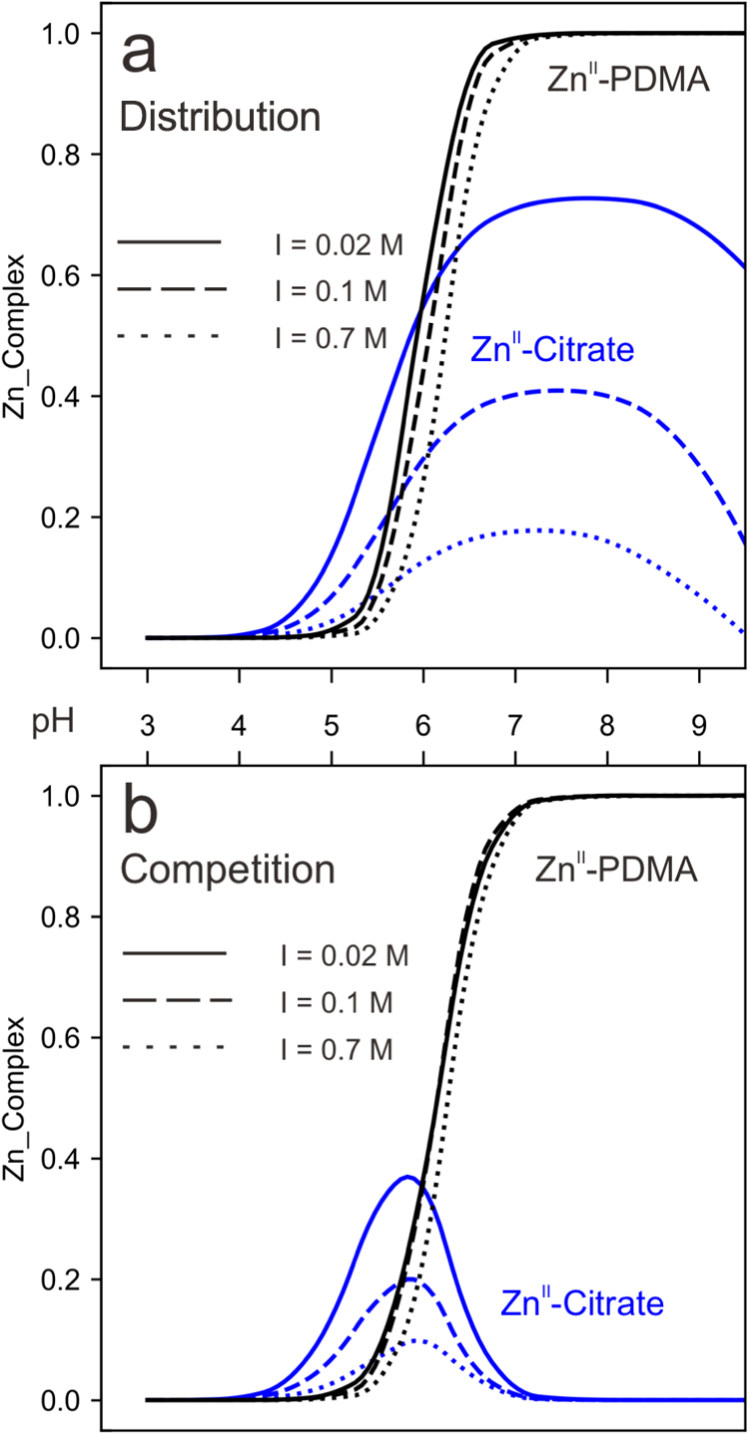
Fraction of Zn^II^ complexed with PDMA and citrate as a function of pH in single ligand (a) and mixed ligand (b) solutions in NaCl solution at different ionic strengths of *I* = 0.02, 0.1, and 0.7 mol/dm^3^. The concentrations used are 10^–6^ mol/dm^3^ for Zn^II^ and 10^–5^ mol/dm^3^ for PDMA and citrate. Different types of lines represent different ionic strengths.

Figure 6a shows the pH-dependent formation of Zn^II^ complexes with PDMA and citrate in low-, medium-, and high-ionic-strength solutions. In low-ionic-strength solutions, the formation of Zn^II^–citrate starts at pH 3.5 and increases to a maximum of 73% at pH 8. In medium- and high-ionic-strength solutions, the onset of formation of Zn^II^–citrate complexes shifts to pH 4.5, reaching no more than 41 and 18% at neutral pH. The increase in ionic strength affects the formation of Zn^II^–citrate complexes significantly.

Figure 6b shows competition between citrate and PDMA for Zn^II^ when both ligands are present at equal concentrations (molar ratio of Zn^II^/PDMA:citrate is 1:10:10). Under these conditions, in neutral and alkaline pH, almost all the Zn^II^ is complexed to PDMA as the log *K*_ML_ value for PDMA is higher than that for citrate (11.48 ± 0.02 and 4.885 ± 0.005, respectively^23^). At weakly acidic to neutral pH (5 to 7), citrate complexes the free Zn^II^ in solution. The maximum complexation occurs at pH 6, with 35, 19, and 10% complexation at low, medium, and high ionic strengths, respectively. These results indicate that competition between PDMA and citrate is unlikely. Instead, a synergistic cooperation between the two ligands is suggested within the pH range of 5–7. This implies that the presence of citrate and PDMA may enhance zinc acquisition by rice, effectively covering a broader soil pH range, which is in agreement with previous studies.^23^ Finally, we assessed the competition of malate, oxalate, and DFOB with PDMA for complexation with Zn^II^ (Supporting Information, Figure S5) when ligands are present at equal concentrations (molar ratio of Zn^II^/PDMA:(malate/oxalate/DFOB) is 1:10:10). Malate, oxalate, and desferrioxamine B (DFOB) do not compete with PDMA (Figure S5). While DFOB is a strong chelator, its log *K*_ML_ value (9.91 ± 0.02^23^) is lower than that of PDMA (11.48 ± 0.02), explaining the lack of competition for Zn^II^ in the presence of PDMA.

### 3.3 Investigation of PDMA Speciation and Zn Uptake in Rice

Having investigated the effects of chemical solution parameters on the formation of M^II,III^–PDMA complexes in electrolyte solutions, we then assessed PDMA speciation in real soil solutions and compared it to zinc uptake observed in rice, using data from a recently conducted pot trial by Suzuki and co-workers.^15^ In that study, rice plants (*O. sativa*, var. Nipponbare) were transplanted into calcareous soil with a pH of 9. 14 days after transplantation, free PDMA and PDMA complexed with Zn^II^ were added to the soil as a fertilizer. The soil solution had a positive redox potential (Eh = +350 mV). Soils were irrigated 3 times a week, and rice plants were harvested 70 days after transplantation. Data pertaining to the experiments, i.e., metal ion concentrations, redox conditions, and ionic strength, are given in Table S2 and in the Materials and Methods Section.

**Figure 7:**
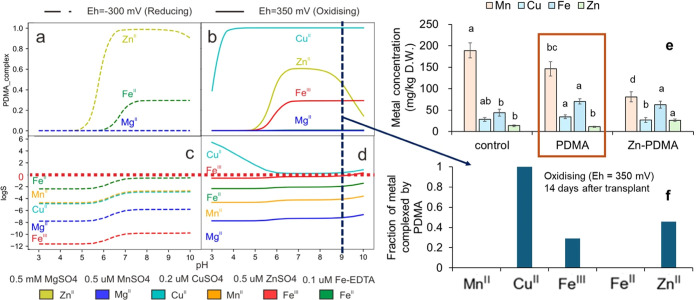
(a, b) Fraction of PDMA complexed with Zn^II^, Fe^II^, Fe^III^, Cu^II^, Mg^II^, and Mn^II^ and (c, d) log of the selectivity ratio (log *S*) as a function of pH in 0.1 mol/dm^3^ NaCl solutions with different redox potentials (a, c): Eh = −300 mV; (b, d): Eh = +350 mV. (e) Metal concentration in rice following the pot experiment performed by Suzuki et al.^15^ Plants were harvested after 70 days from transplant. Shown are metal concentrations (Mn, Cu, Zn, Fe) in control, after addition of free PDMA, and Zn^II^–PDMA. Fraction of PDMA with Zn^II^, Fe^II^, Fe^III^, Cu^II^, and Mn^II^ in the soil solution of the experiment performed by Suzuki et al.^15^ at the time of PDMA application (f). The concentrations of metal ions and PDMA used in the model solution are based on the values reported by Suzuki et al.^15^ (see the Materials and Methods Section).

pH-dependent formation of M^II,III^–PDMA complexes and the selectivity of Zn^II^ for PDMA in the soil solution under oxidizing (Eh = +350 mV) and reducing (Eh = – 300 mV) conditions are shown in Figure 7a–d. In solutions with positive redox potential, we find significant formation of PDMA complexes with Cu^II^ between pH 4 and 10, with Zn^II^ between pH 6.5 and 8, and with Fe^III^ between pH 6.5 and 10. Zn^II^ complexation rapidly decreases above pH 8 due to the competition of Fe^III^. The selectivity for Cu^II^ decreases above pH 6 and increases above pH 9 along with that of Fe^III^, emphasizing the preferred complexation of copper and iron in alkaline pH in oxidized solutions. In solutions with negative redox potential (i.e., at the point of harvest), PDMA is quantitatively complexed to Zn^II^ in the pH range between 6 and 10 (Figure 7a), and the log *S* is negative for all metals, indicating that PDMA primarily coordinates with Zn^II^ with minimal competition from any of the other metal ions.

Figure 7f displays the formation of PDMA complexes with Zn^II^, Fe^III^, Fe^II^, Mn^II^, and Cu^II^ in the soil solution following the application of PDMA as a fertilizer 14 days after transplantation (Eh = +350 mV, pH = 9). Figure 7e shows Mn, Cu, Fe, and Zn concentrations in the rice plants at harvest time, during trials when PDMA was applied as free PDMA and as the Zn^II^–PDMA complex. We find that in solution with positive redox potential, PDMA formed complexes preferentially with Cu^II^ and Fe^III^ and that Zn uptake increased when PDMA was added as the Zn^II^–PDMA complex but not when it was added as the free PDMA ligand. We conclude that to increase Zn^II^ uptake in oxidized conditions, fertilization with free PDMA is not recommended due to high competition with Cu^II^ and Fe^III^; on the other hand, Zn^II^–PDMA application is recommended because, once formed, this complex is stable and increases the uptake of Zn in rice. Furthermore, we suggest applying free PDMA in reduced conditions and alkaline pH to facilitate the formation of the [Zn(PDMA)]^−^ complex.

### 3.4 Effect of Excess Zn, Cu, and Fe on Formation of Zn^II^–PDMA Complexes

In the last step, we determined the effect of excess Zn, Cu, and Fe on the formation of Zn^II^–PDMA complexes in reducing and oxidizing solutions. This information is critical if PDMA is to be used as a fertilizer due to the high log *K*_ML_ values of Cu and Fe with PDMA. Copper and iron concentrations in soil solutions vary significantly due to the soil parent material and anthropogenic activities, i.e., fertilizer addition to the soil surface.^26−28^ To this end, we assessed first the formation of Zn^II^–PDMA with Cu^II^ and Fe^III^ in excess (between 2 and 50 times, see Table 3a) and then with Zn^II^ in excess (between 2 and 10 times, see Table 3b). We investigated reducing (Eh = −300 mV) and oxidizing (Eh = +350 mV) solutions at pH 5, 7, and 9.

**Table 3:**
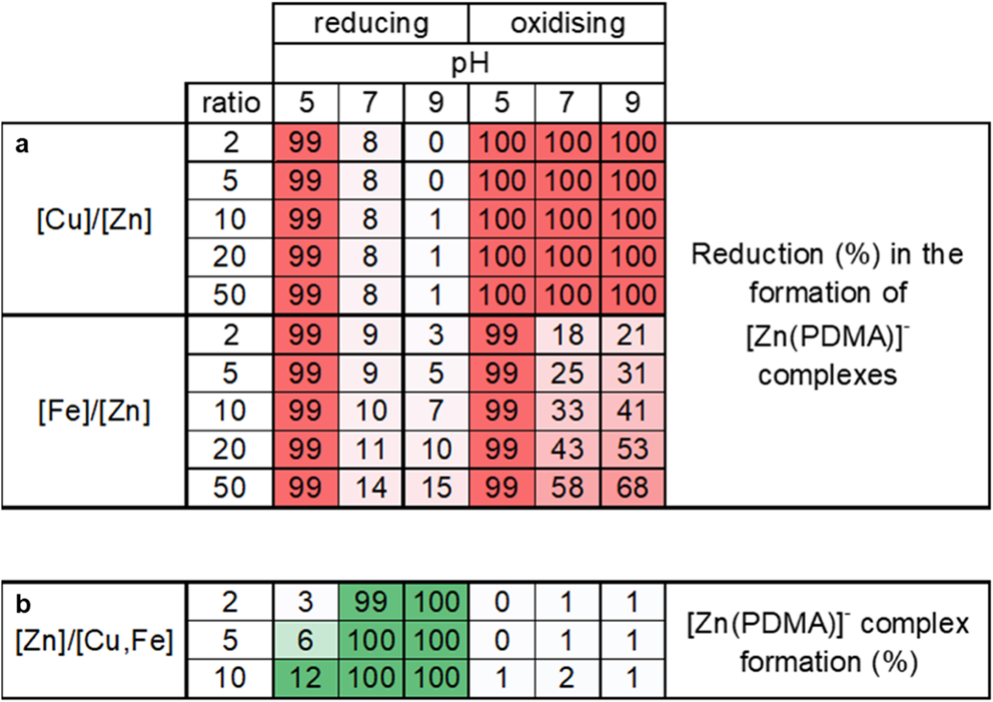
Effect of (a) Excess Cu and Fe, Expressed Using the [(Cu,Fe)]/[Zn] Ratio, on the Reduction of [Zn(PDMA)]^−^ and (b) Excess Zn, Expressed Using the [Zn]/[(Fe,Cu)] Ratio, on the Formation of [Zn(PDMA)]^−^a

a Solutions consisted of 0.1 mol/dm^3^ NaCl and pH 5, 7, and 9 under reduced and oxidized conditions. PDMA was present in excess with a concentration of 10^–6^ mol/dm^3^. Initial metal concentrations were based on literature data.^28,42^ Distinct color shadows represent the increment (from light to dark green) and the reduction (from light to dark red) percentage of [Zn(PDMA)]^−^ complex formations.

Table 3a shows the effect of excess Cu and Fe, ranging between 2 and 50 times, on the formation of Zn^II^–PDMA complexes in reducing and oxidizing soil solutions with an initial Zn^II^/(Cu^II^, Fe^III^) ratio of 0.01. Under reducing conditions, Zn^II^–PDMA formation is lowered most significantly at pH 5, with a 99% reduction when Cu^II^ and Fe^III^ are in excess. At pH 7, the effect on the formation of Zn^II^–PDMA is less significant, i.e., 8% reduction when Cu^II^ is in excess and between 9 and 14% reduction when Fe^III^ is in excess. At pH 9, reduction is down to 1% if Cu^II^ is 50 times in excess and between 3 and 15% if Fe^III^ is in excess. Under oxidizing conditions, if Cu^II^ is in excess, Zn^II^–PDMA formation is 100% reduced across the entire pH range studied. If Fe^III^ is in excess, Zn^II^–PDMA formation decreases by 99% at pH 5, between 18 and 58% at pH 7, and between 21 and 68% at pH 7.

Table 3b shows the effect of excess Zn ranging between 2 and 10 times on the formation of Zn^II^–PDMA complexes. Under reducing conditions, in solutions at pH 5, between 3 and 12% of Zn^II^–PDMA complexes are formed if zinc is in excess between 2 and 10 times. In solutions at pH 7 and 9, the formation of Zn^II^–PDMA complexes increases significantly and ranges between 99 and 100% if Zn^II^ is in excess between 2 and 10 times (Table 3b). Under oxidizing conditions, in contrast, Zn^II^–PDMA complexes are formed at most 2% in solutions with pH 7 and 10 times in zinc excess. [Cu(PDMA)]^−^ is the dominant complex across all pH values ranging from 95 to 100%. Iron is present in its divalent form within the acidic pH range of the solutions studied^25^ and hence cannot compete with Zn^II^ and Cu^II^ for PDMA complexation. As the pH increases, iron precipitates as Fe_2_O_3_.^25,46^

In conclusion, our results show that PDMA is a strong chelator, forming stable complexes with Zn^II^ between pH 6 and 9, and its behavior in aqueous solutions is similar to natural bioavailable ligands such as DMA. To optimize the fertilization efficacy, PDMA should be applied in flooded soil at pH between 7 and 9, where competition with Cu^II^ and Fe^III^ is minimized, or precomplexed Zn^II^–PDMA fertilizer should be applied in both flooded and aerated soil. In this way, PDMA should be able to significantly reduce zinc deficiency in soil. Future work will now focus on evaluating these boundary conditions in a new set of plant uptake experiments.

## Data Availability

The data sets generated and/or analyzed during the current study are available on Zenodo, https://zenodo.org/uploads/12657554

## Supporting Information Available

Affinity constants (log *K*_ML_) for the formation of 1:1 metal complexes with ligands from the mugineic acid family (MA, DMA, and HMA) and with PDMA (Table S1); additional details for the PHREEQC model (Table S2); chemical structures of mugineic acid family (MA) ligands (Figure S1); speciation diagrams for PDMA and metal–PDMA systems calculated using HySS (Figure S2); manual fitting of titration results for PDMA and metal–PDMA systems using Hyperquad (Figure S3); correlation between Zn^II^–PDMA speciation modeled using HySS and PHREEQC (Figure S4); fraction of Zn^II^ complexed by PDMA in the presence of oxalate, malate, and DFOB (Figure S5); fraction of Zn^II^ and Ni^II^ complexed by PDMA under varying pH, ionic strengths, and redox conditions (Figure S6); and metal–ligand complex formation in aqueous solutions at different Zn/(Fe,Cu) ratios, pH, and redox potential modeled using PHREEQC (Figure S7) (PDF)
