# Supplementary material for: Enhancing Zinc Bioavailability in Rice Using the Novel Synthetic Siderophore Ligand Proline-2′-Deoxymugineic Acid (PDMA): Critical Insights from Metal Binding Studies and Geochemical Speciation Modeling
Source: J Agric Food Chem. 2025 Mar 27;73(14):8243–53. doi: 10.1021/acs.jafc.5c02128 (PMC11987015; doi:10.1021/acs.jafc.5c02128)
Supplement: Supplementary file 1 — jf5c02128_si_001.pdf [file jf5c02128_si_001.pdf]

**Supplementary Information**

# Enhancing zinc bioavailability in rice using the novel synthetic siderophore ligand proline-2'-deoxymugineic acid (PDMA): Critical insights from metal binding studies and geochemical speciation modelling

Claudia Rocco [a,b]*, Motofumi Suzuki [c], Ramon Vilar [d], Enrique Garcia-España [e], Salvador Blasco [e], Gerald Larrouy-Maumus [a], Colin Turnbull [a], Matthias Wissuwa [f], Xuan Cao [b], and Dominik Weiss [b]*

[a] Centre for Bacterial Resistance Biology, Department of Life Sciences, Faculty of Natural Sciences, Imperial College London, London, SW7 2AZ, United Kingdom

[b] Department of Earth Science and Engineering, Imperial College London, South Kensington Campus, London, SW7 2AZ, United Kingdom

[c] Aichi Steel Corporation, Tokai-shi, Aichi 476-0003, Japan

[d] Department of Chemistry, Imperial College London, White City Campus, London, W12 0BZ, United Kingdom

[e] Instituto de Ciencia Molecular (ICMol), University of Valencia, C/Catedrático José Beltrán Martínez, 2, Paterna 46980, Spain

[f] Institute of Crop Science and Resource Conservation (INRES), University of Bonn, Karl Robert-Kreiten-Strasse 13, Bonn 53115, Germany

*** Corresponding authors:**

Email: c.rocco@imperial.ac.uk

Email: d.weiss@imperial.ac.uk

**Table S1.** Affinity constants ($logK_{ML} = log\beta_{110}$) for biologically relevant 1:1 complexes ($[M^{II}:L]$ or $[M^{III}:L]$) of ligands from the mugineic acid family (MA, DMA and HMA) and PDMA taken from the literature (Murakami et al., 1989; Evers et al., 2024; Suzuki et al., 2021). HMA = 3-epi-hydroxymugineic acid, DMA = 2′deoxymugineic acid and MA = mugineic acid, PDMA = Proline-2`-deoxymugineic acid.

| **Metal** | **Ligand ($logK_{ML}$)** | | | | |
|---|---|---|---|---|---|
| | DMA[1] | MA[1] | HMA[1] | PDMA[2] | PDMA[3] |
| Zinc$^{II}$ | 12.84±0.06 | 12.69±0.02 | 11.44±0.04 | 14.85±0.1 | - |
| Iron$^{II}$ | 10.45±0.04 | 10.14±0.06 | 10.02±0.08 | - | - |
| Iron$^{III}$ | 18.38±0.03 | 17.71±0.02 | - | - | 17.1 |
| Copper$^{II}$ | 18.70±0.01 | 18.10±0.04 | 17.92±0.01 | 19.5±0.7 | - |
| Nickel$^{II}$ | 14.78±0.01 | 14.92±0.01 | 14.39±0.04 | 11.8±0.4 | - |
| Manganese$^{II}$ | 8.29±0.02 | 8.30±0.04 | 8.03±0.01 | 7.6±0.2 | - |
| Cobalt$^{II}$ | - | - | - | 13.8±0.4 | - |

[1] Murakami et al., 1989; [2] Evers et al., 2024; [3] Suzuki et al., 2021

**Table S2.** Complementary details regarding the solution condition used in the PHREEQC model for all investigated metal ions at different ionic strengths and redox potential

| **Experiment** | **Parameter evaluated** | **Conditions** | **Concentrations (mol dm$^{-3}$)** |
|---|---|---|---|
| 1 | Salinity | Excess of PDMA<br>I = 0.02, 0.1, 0.7 mol/dm$^3$ | PDMA = $10^{-5}$<br>$Zn^{II}$ = $10^{-6}$ |
| 2 | | Excess of PDMA<br>I = 0.02, 0.1, 0.7 mol/dm$^3$ | PDMA = $10^{-5}$<br>$Fe^{II,III}$ = $10^{-6}$ |
| 3 | | Excess of PDMA<br>I = 0.02, 0.1, 0.7 mol/dm$^3$ | PDMA = $10^{-5}$<br>$Ni^{II}$ = $10^{-6}$ |
| 4 | | Excess of PDMA<br>I = 0.02, 0.1, 0.7 mol/dm$^3$ | PDMA = $10^{-5}$<br>$Cu^{II}$ = $10^{-6}$ |
| 5 | Micronutrient competition | Excess of PDMA<br>I = 0.1 mol/dm$^3$ | PDMA = $10^{-5}$<br>M = $10^{-6}$ |
| 6 | | Nutrient solution<br>Eh = +350 mV<br>Eh = -300 mV | |

**Figure S1.** Chemical structures of naturally occurring mugineic acid family (MA) ligands. Nicotianamine (NA), 2'- deoxymugineic acid (DMA), mugineic acid (MA) and 3-hydroxymugineic acid (HMA).

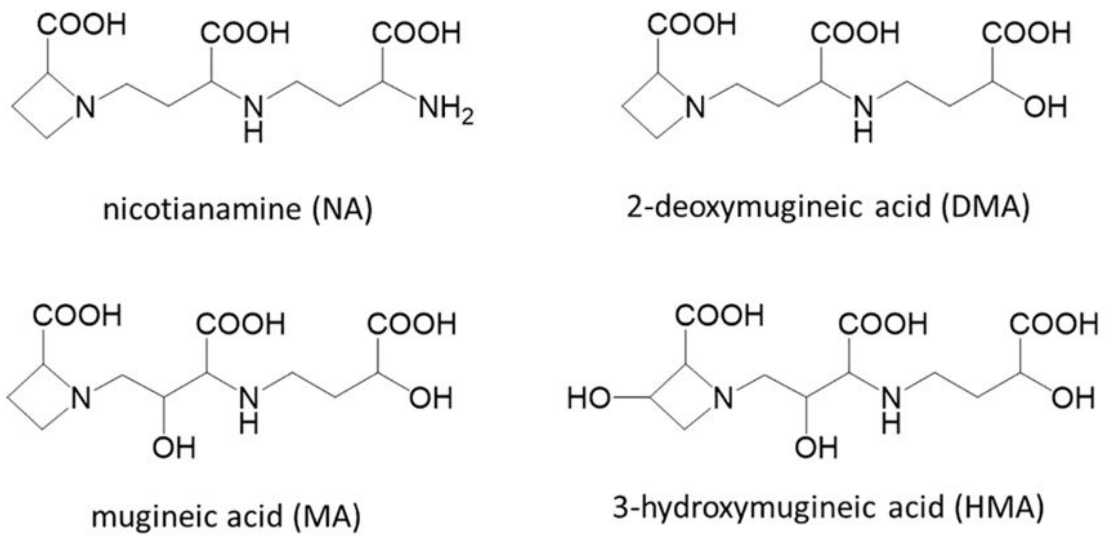

**Figure S2.** Speciation diagrams of PDMA and various metal-PDMA systems

(a) PDMA = [L] = 6.7 x $10^{-4}$ mol/$dm^3$,
(b) $Zn^{II}$:L = 1:1, [$Zn^{II}$] = 6.0 x $10^{-4}$ mol/$dm^3$and [L] = 6.7 x $10^{-4}$ mol/$dm^3$,
(c) $Fe^{II}$:L = 1:1, [$Fe^{II}$] = 6.0 x $10^{-4}$ mol/$dm^3$ and [L] = 6.7 x $10^{-4}$ mol/$dm^3$,
(d) $Cu^{II}$:L = 1:1, [$Cu^{II}$] = 6.0 x $10^{-4}$ mol/$dm^3$ and [L] = 6.7 x $10^{-4}$ mol/$dm^3$.

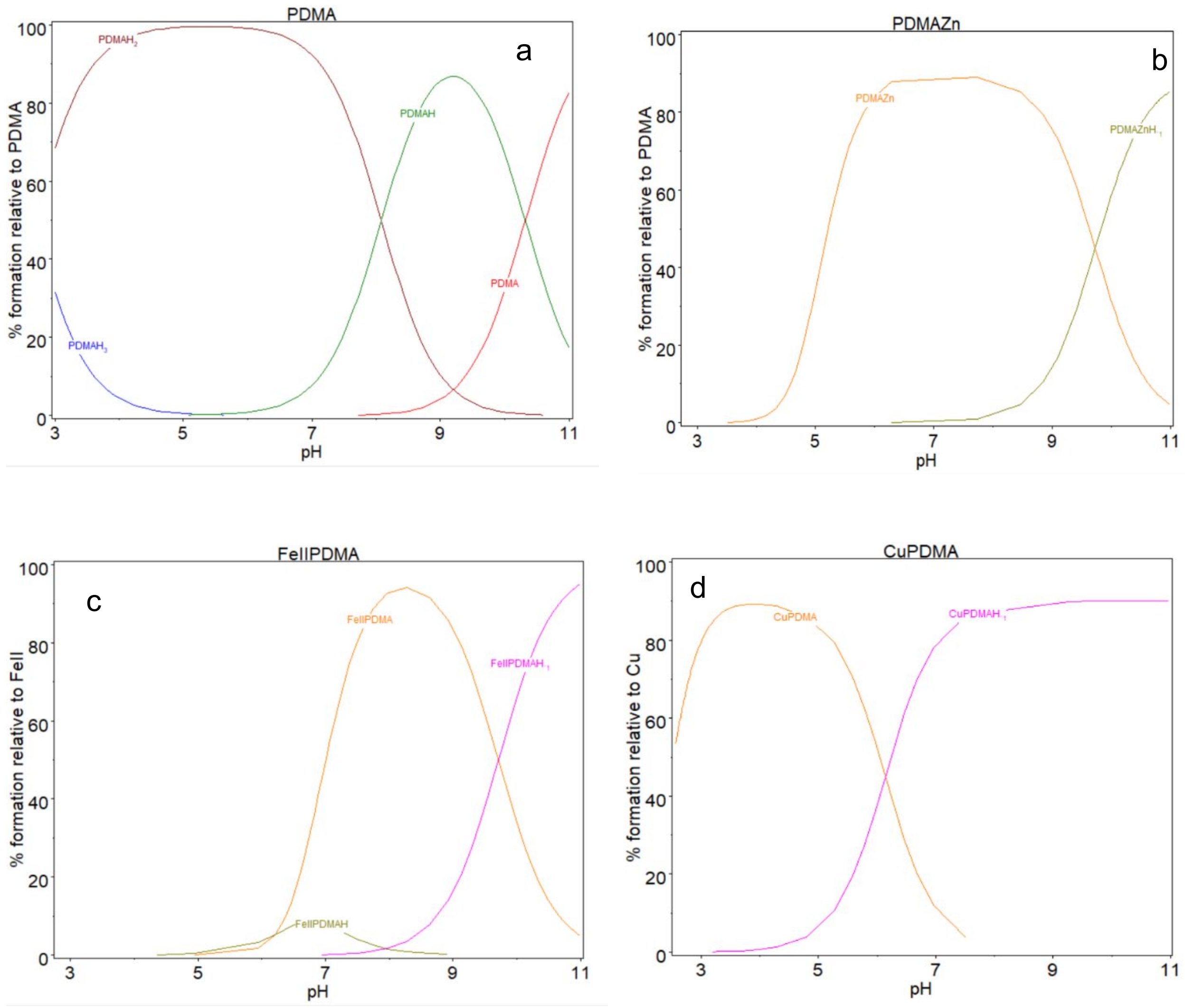

**Figure S3**. Manual fitting of potentiometric data following titrations of PDMA and metal-PDMA systems at I = 0.15 mol/dm$^3$ NaCl and T = 298.1 ± 0.1 K using [PDMA] = 6.7 x 10$^{-4}$ mol/dm$^3$ and metal$^{II}$:PDMA = 1:1, [M] = 6.0 x 10$^{-4}$ mol/dm$^3$ and [PDMA] = 6.7 x 10$^{-4}$ mol/dm$^3$.

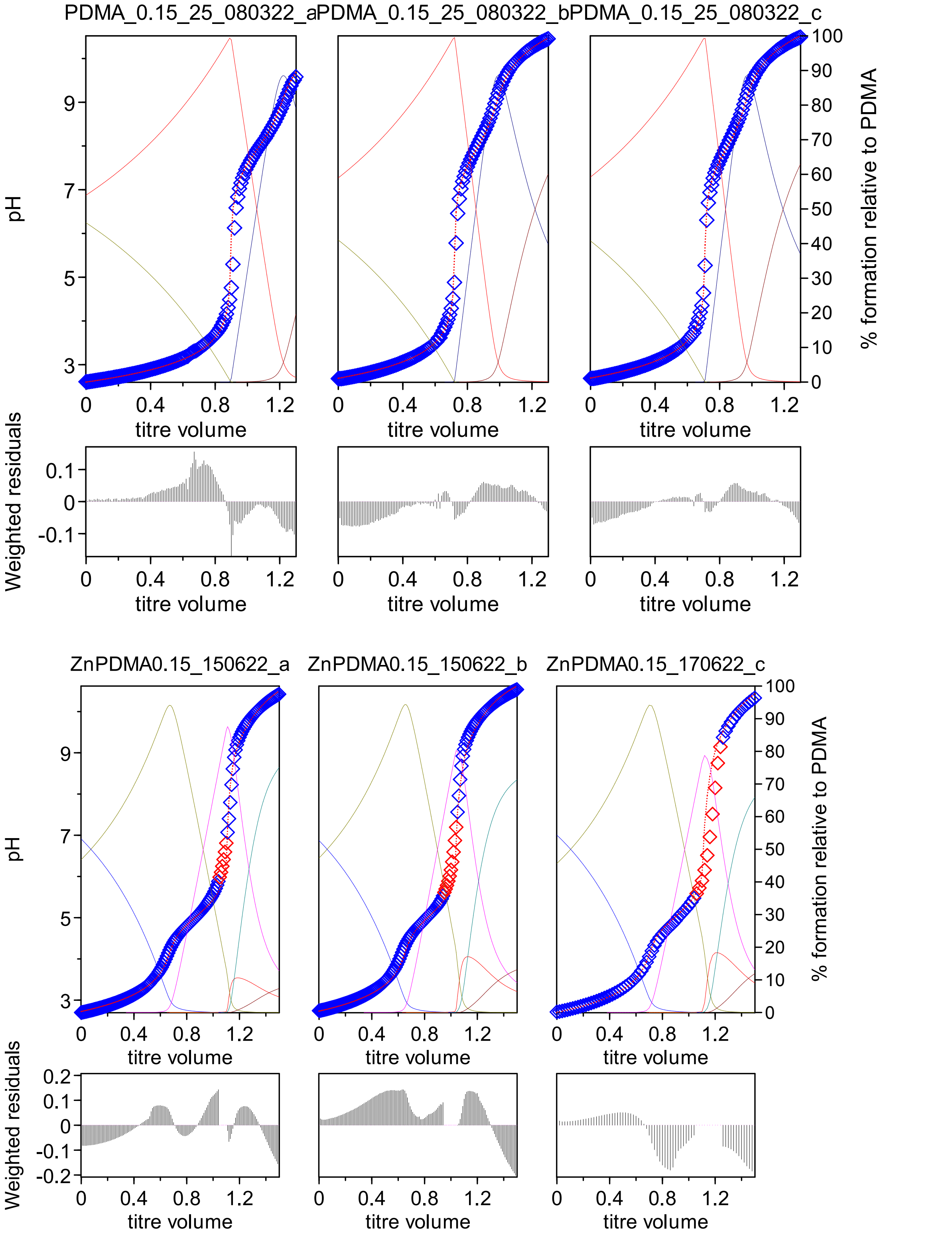

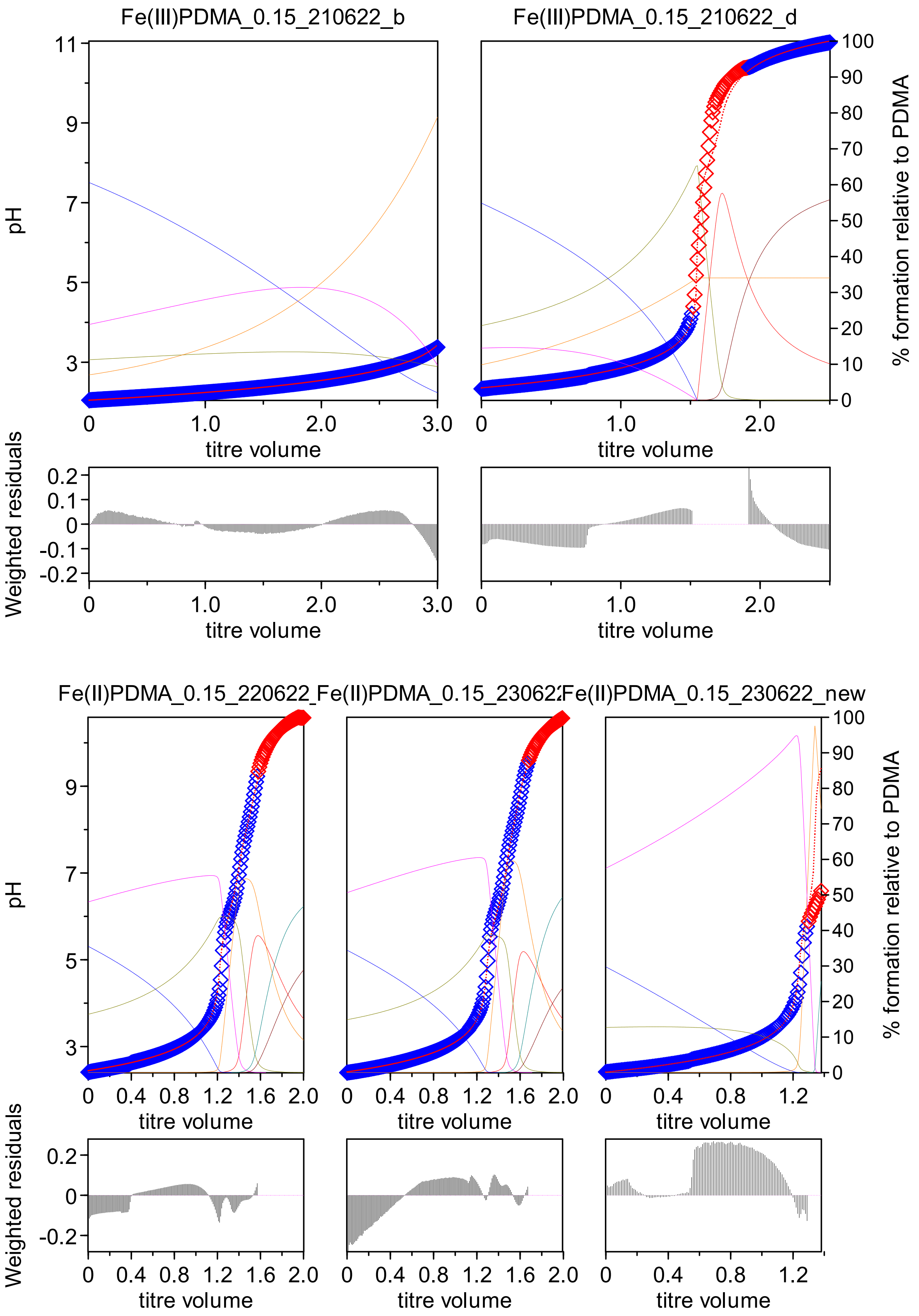

Fe(III)PDMA_0.15_210622_b
Fe(III)PDMA_0.15_210622_d
pH
% formation relative to PDMA
titre volume
Weighted residuals
Fe(II)PDMA_0.15_220622
Fe(II)PDMA_0.15_23062
Fe(II)PDMA_0.15_230622_new

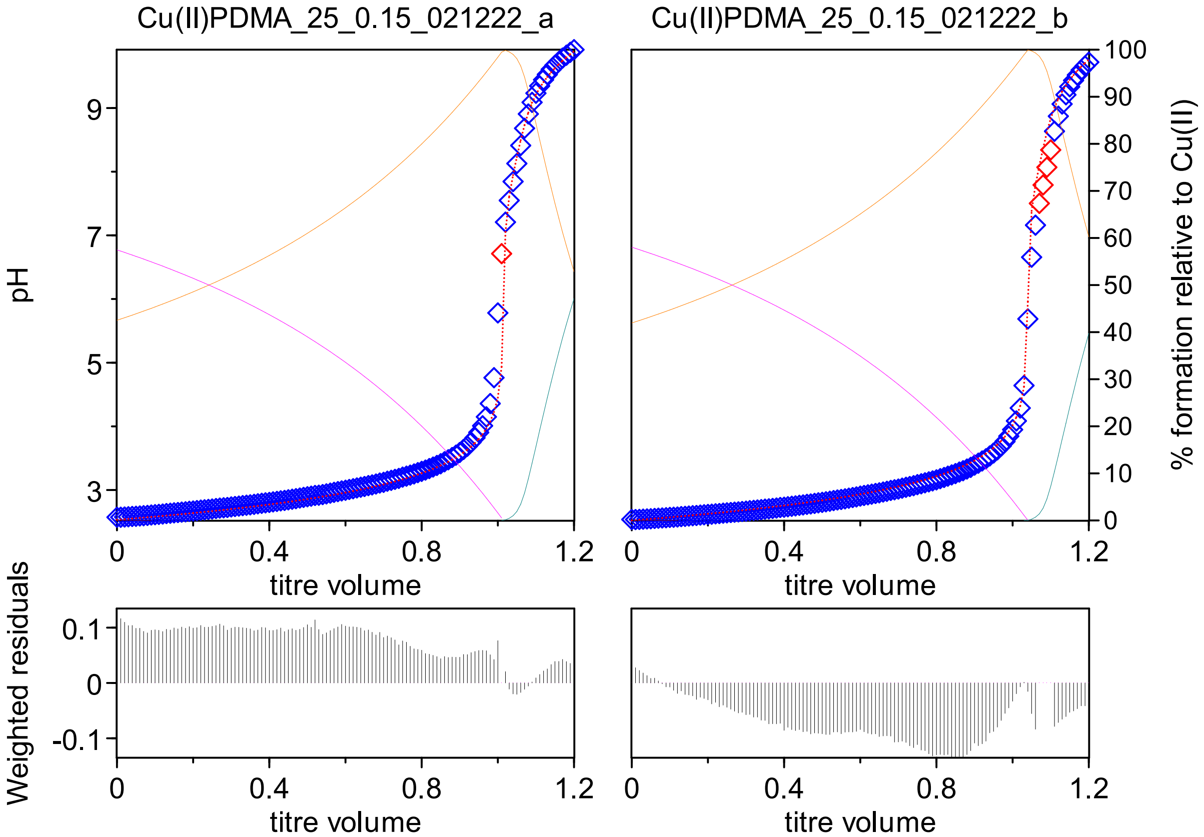

Cu(II)PDMA_25_0.15_021222_a
Cu(II)PDMA_25_0.15_021222_b
pH
% formation relative to Cu(II)
titre volume
Weighted residuals

**Figure S4.** $Zn^{II}$-PDMA speciation diagrams obtained in 0.1 mol/$dm^3$ NaCl solutions modelled using HySS and PHREEQC and their correlation

(a) $Zn^{II}$-PDMA in HySS = [L] = 1:10, $[Zn^{II}] = 10^{-6}$ mol/$dm^3$ and $[L] = 10^{-5}$ mol/$dm^3$
(b) $Zn^{II}$-PDMA in PHREEQC = 1:10, $[Zn^{II}] = 10^{-6}$ mol/$dm^3$ and $[L] = 10^{-5}$ mol/$dm^3$
(c) Correlation between HySS and PHREEQC model.

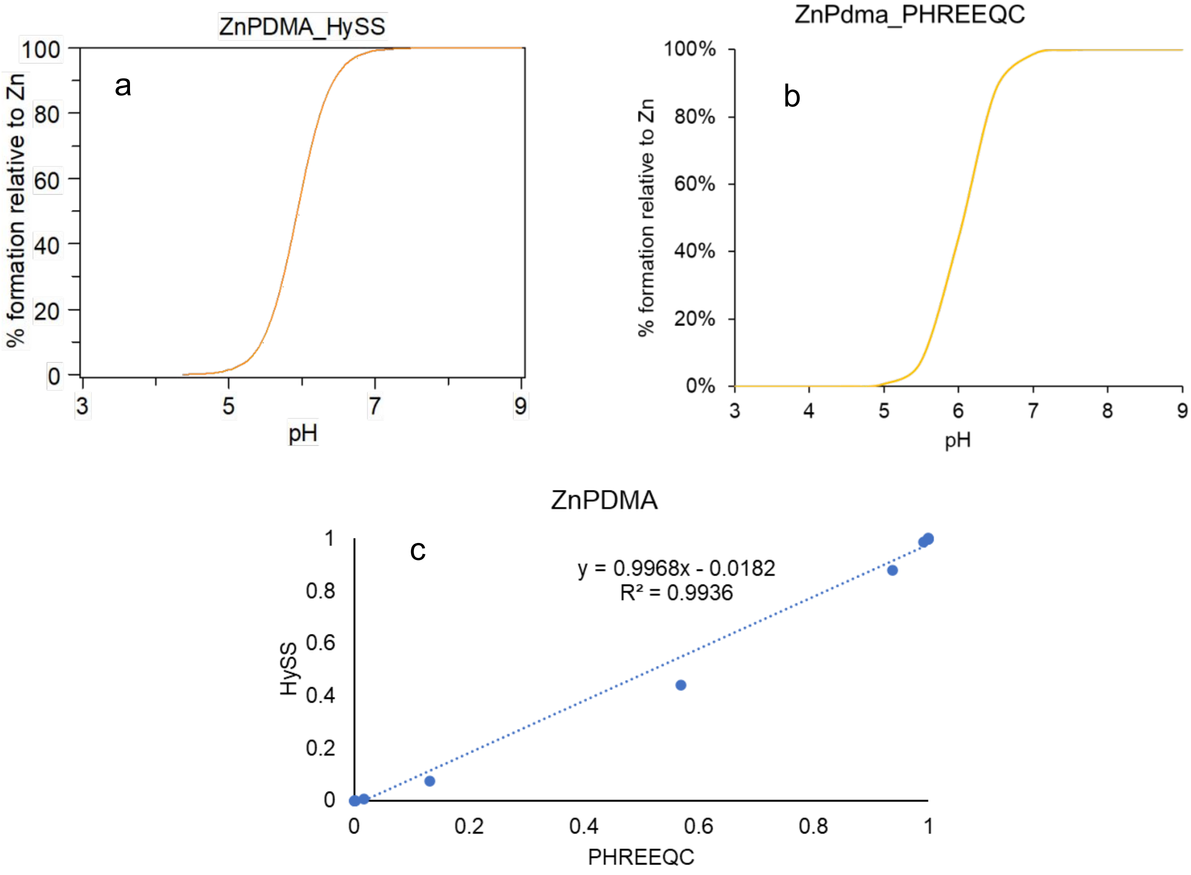

**Figure S5.** Fraction of $Zn^{II}$ complexed by PDMA in presence of oxalate (a), malate (b) and DFOB (c: modelled using PHREEQC; d: modelled using HySS).

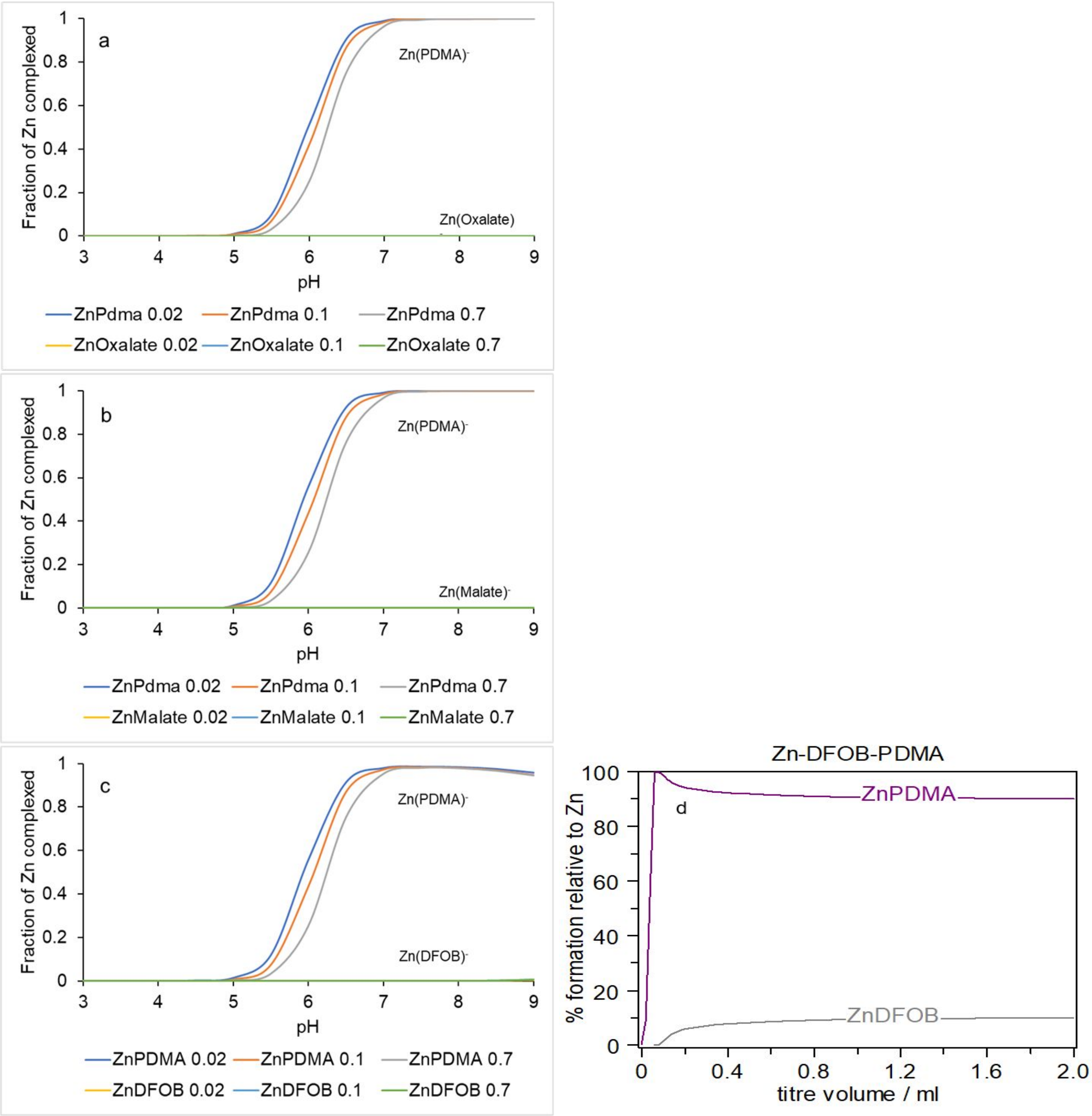

**Figure S6.** Fraction of $Zn^{II}$ and $Ni^{II}$ complexed with PDMA in solutions with different pH, ionic strength and redox conditions. a: $Zn^{II}$ and -300 mV; b: $Zn^{II}$ and +350 mV; c: $Ni^{II}$ and -300 mV; d: $Ni^{II}$ and+350 mV.

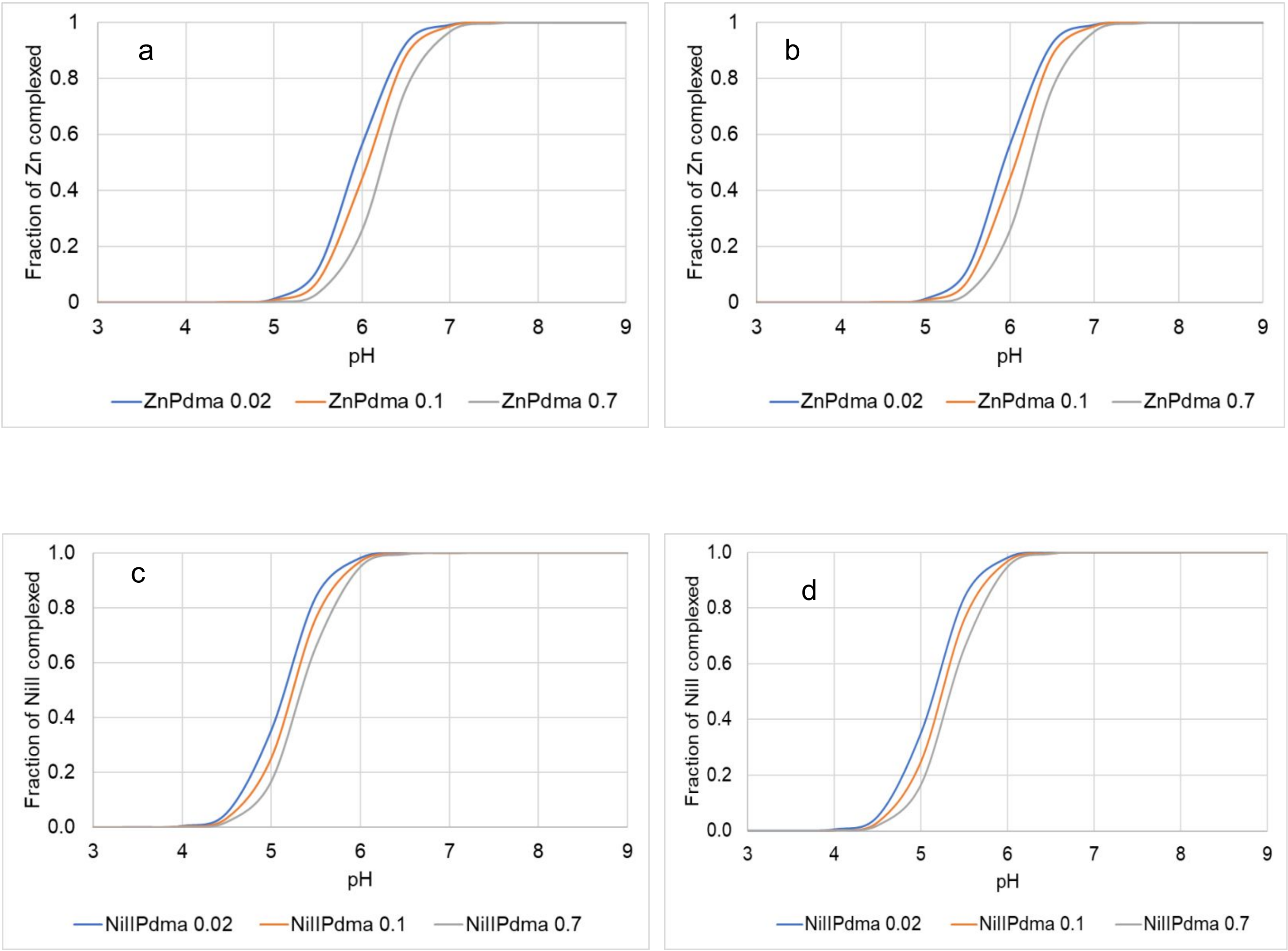

**Figure S7.** Percent metal-ligand complexes formed in 0.1 mol/dm$^3$ NaCl solution with different Zn/($Fe^{III}$;$Cu^{II}$) ratios, pH, and redox potentials. a-b-c: represent Zn/($Fe^{III}$;$Cu^{II}$) ratio typically found in soil at Eh = -300 mV; d-e-f: represent Zn/($Fe^{III}$;$Cu^{II}$) ratios referred to Zn fertilization at Eh = -300 mV; g-h-i represent Zn/($Fe^{III}$;$Cu^{II}$) ratio typically found in soil at Eh = +350 mV; l-m-n: represent the Zn/($Fe^{III}$;$Cu^{II}$) ratios referred to Zn fertilization at Eh = +350 mV.

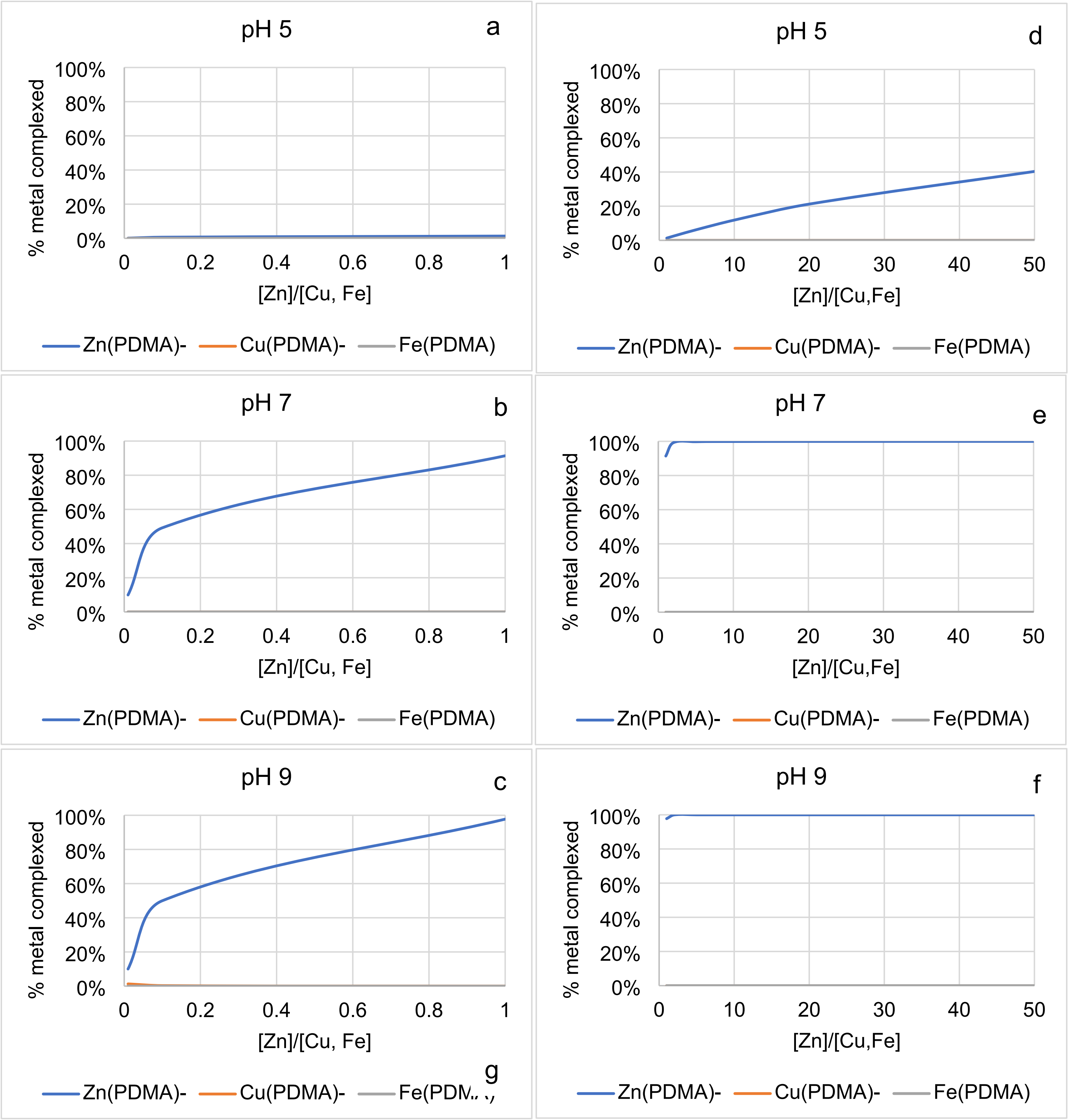

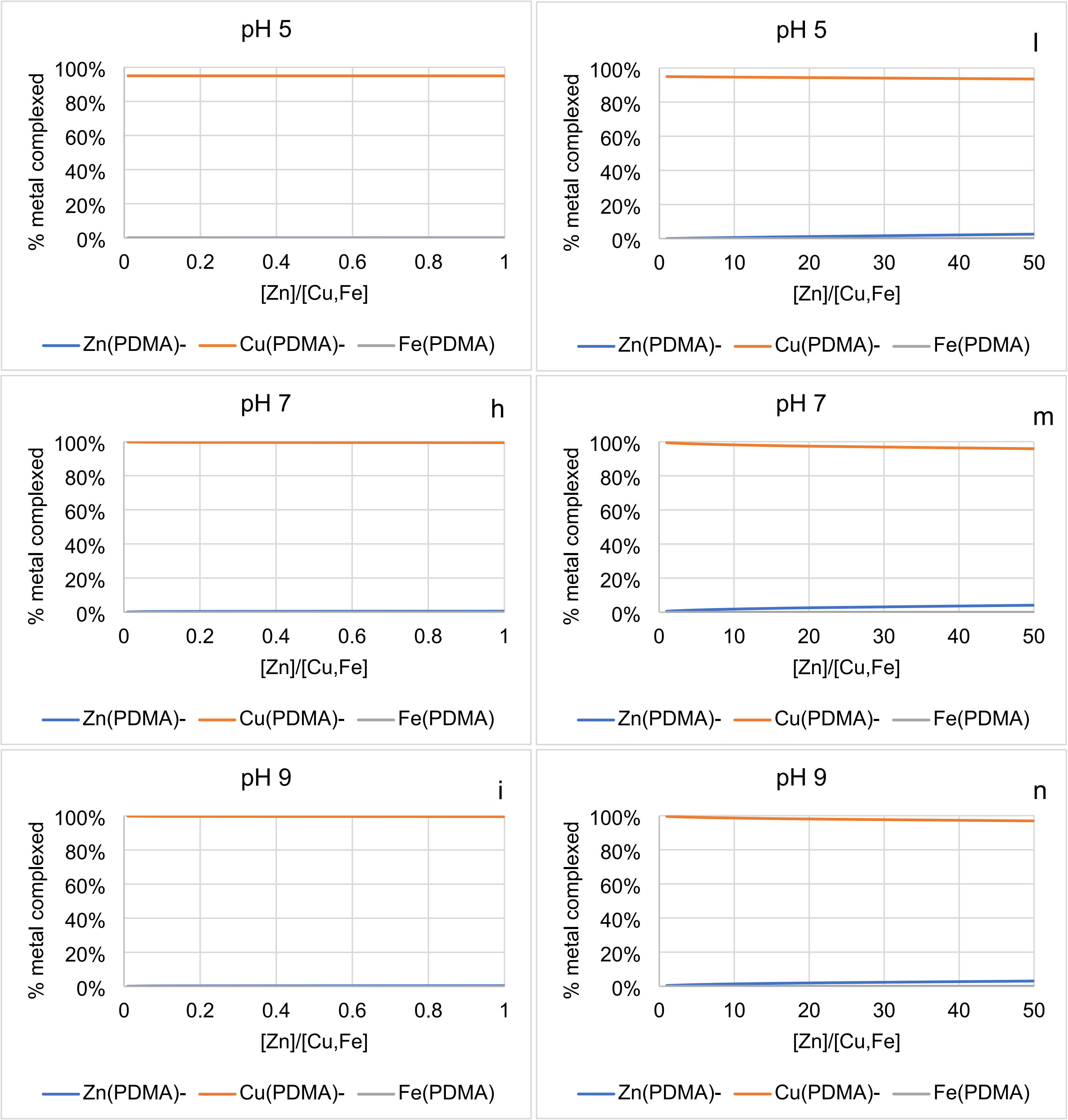
pH 5
% metal complexed
100%
80%
60%
40%
20%
0%
0
0.2
0.4
0.6
0.8
1
[Zn]/[Cu,Fe]
Zn(PDMA)-
Cu(PDMA)-
Fe(PDMA)
pH 5
l
10
20
30
40
50
pH 7
h
pH 7
m
pH 9
i
pH 9
n
